# Exploring culturally acceptable, nutritious, affordable and low climatic impact diet for Japanese diets: proof of concept of applying a new modelling approach using data envelopment analysis

**DOI:** 10.1017/S0007114522000095

**Published:** 2022-12-28

**Authors:** Minami Sugimoto, Elisabeth H. M. Temme, Sander Biesbroek, Argyris Kanellopoulos, Hitomi Okubo, Aya Fujiwara, Keiko Asakura, Shizuko Masayasu, Satoshi Sasaki, Pieter van’t Veer

**Affiliations:** 1 Department of Social and Preventive Epidemiology, Division of Health Sciences and Nursing, Graduate School of Medicine, University of Tokyo, Tokyo 113-0033, Japan; 2 The National Institute for Public Health and the Environment (RIVM), Bilthoven, the Netherlands; 3 Division of Human Nutrition and Health, Wageningen University, P.O. Box 176700 AA Wageningen, the Netherlands; 4 Operations Research and Logistics group, Wageningen University, 6706 KN Wageningen, the Netherlands; 5 Department of Health Promotion, National Institute of Public Health, Saitama, Japan; 6 Department of Nutritional Epidemiology and Shokuiku, National Institute of Biomedical Innovation, Health and Nutrition, 1-23-1 Toyama Shinjuku-ku, Tokyo 162-8636, Japan; 7 Department of Environmental and Occupational Health, School of Medicine, Toho University, Tokyo 143-8540, Japan; 8 Ikurien-Naka, Ibaraki 311-0105, Japan; 9 Department of Social and Preventive Epidemiology, School of Public Health, University of Tokyo, Tokyo 113-0033, Japan

**Keywords:** Sustainable diets, Japanese, Data envelopment analysis, Diet model

## Abstract

A future sustainable dietary pattern for Japanese is yet undefined. This study aimed to explore more sustainable Japanese diets that are nutritious, affordable and with low greenhouse gas emissions (GHGE) and particular emphasis on cultural acceptability. A newly developed data envelopment analysis (DEA) diet model was applied to 4-d dietary record data among 184 healthy Japanese men and 185 women volunteers aged 21–69 years. Alternative diets were calculated as the linear combinations of observed diets. Firstly, for each individual, four modelled diets were calculated that maximised cultural acceptability (i.e. minimise dietary change from observed diet), maximised nutritional quality assessed by the Nutrient-Rich Food Index (NRF), minimised monetary diet costs or minimised diet-related GHGE. The final modelled diet combined all four indicators. In the first four models, the largest improvement was obtained for each targeted indicator separately, while relatively small improvements or unwanted changes were observed for other indicator. When all indicators were aimed to optimise, the NRF score and diet-related GHGE were improved by 8–13 % with the lower monetary cost than observed diets, although the percentage improvement was a bit smaller than the separate models. The final modelled diets demanded increased intakes for whole grains, fruits, milk/cream/yogurt, legumes/nuts, and decreased intakes for red and processed meat, sugar/confectioneries, alcoholic and sweetened beverages, and seasonings in both sexes. In conclusion, more sustainable dietary patterns considering several indicators are possible for Japanese, while total improvement is moderate due to trade-offs between indicators and methodological limitation of DEA diet model.

Under the rising concern on climate change, the concept of ‘sustainable diets’ has been defined as ‘protective and respectful of biodiversity and ecosystems, culturally acceptable, accessible, economically fair and affordable; nutritionally adequate, safe and healthy; while optimising natural and human resources^(1)^’. Considering that dietary intake drives the food production system, previous studies suggested an urgent need for change in individual diets from several perspectives including health and environmental sustainability^(2,3)^. For the environmental dimension, a number of studies have focused on climatic impact measured using diet-related greenhouse gas emissions (GHGE) given the 15–30 % global contributions from the food sector to total GHGE^(4–7)^.

Recently, optimisation models using mathematical programming techniques have been used for designing sustainable dietary choices^(8–10)^. These models aim to find combinations of food items that minimise an objective function (e.g. the deviation from the current average or each individual observed diet) under a given set of constraints^(8,11–17)^. To avoid unrealistic dietary changes in optimised diets, several acceptability constraints and additional assumptions are defined such as limiting amount on specific food groups consumption, such as allowing consumption between 10th and 90th percentile observed consumption in the whole population^(12–18)^. In individual-based optimisation, it was additionally limited to introduce new foods currently not consumed by the targeted individual into alternative diets^(8,11,16)^. However, even with the application of such constraints, the calculated diets could result in an unrealistic combination of food items. This is because optimised diets were calculated from (non)linear combinations of food items or food groups.

In a recent study, a diet model based on data envelopment analysis (DEA) diet model was proposed as an alternative method of mathematical optimisation models^(19)^. In the application of DEA to dietary modelling, observed diets are benchmarked based on intakes and alternative modelled diets are then calculated as linear combinations of observed diets. The strength of this method is higher feasibility of the modelled diets because it is calculated by combining existing whole diets, that is, combinations of food items. In contrast, DEA diet model limit the degree of change from the observed diets. Thus, DEA diet model could be useful when placing particular emphasis on culturally acceptable diet. In the original DEA diet model, observed diets were benchmarked using twelve nutrients^(19)^ included in the Nutrient-Rich Food Index (NRF) 9.3^(20,21)^. However, the health aspects of the diet might not be sufficiently accounted for by such a limited number of nutrients. To take into account considerable scientific evidence about dietary intake and risk of disease, subsequent studies were proposed using food groups from food-based dietary guidelines (FBDG) to benchmark diets^(22,23)^, as overall indicators of diets that likely reduce risk of non-communicable diseases^(24)^. This diet-based DEA model could be useful to design the alternative dietary patterns that are nutritious, environmentally sustainable and economically affordable, while implicitly considering acceptability aspects of the diet.

Apart from methodological limitations of studies designing alternative diets, the majority of previous studies were from Western countries, where livestock meat largely contributes to diet-related GHGE. Reducing consumption of red meat and dairy products and increasing fruits and vegetables were generally suggested in previous studies to help promote diets with higher nutritional adequacy and lower environmental impact^(13–16,18)^. However, for Asian populations, including Japanese, who consume higher amounts of fish and seafood and legumes, the optimised dietary pattern for sustainable diets might not necessarily to be similar to Western countries. Previously, recommendations for increasing the consumption of meat and alternatives (including eggs, meat and fish) among Japanese young adults were shown by a linear programming optimisation model focusing on nutritional goals^(12)^. Because of the large contribution of fish as well as meat to the diet-related GHGE among Japanese^(25)^, optimised dietary patterns could differ when the climatic impact would be taken into account in the model. Furthermore, only a few studies considered the monetary cost of diets in addition to the environmental indicators in mathematical optimisation of diets^(13–15)^. Moreover, although trade-offs between different sustainable indicators (e.g. nutrient intakes and diet-related GHGE) have been previously suggested^(14,17,18,26,27)^, studies directly investigating trade-offs are sparse. Thus, this study aimed (i) to model the optimised diet in terms of cultural acceptability, nutrient intake, affordability and diet-related GHGE among Japanese adults using DEA diet model and (ii) to investigate trade-offs between the different sustainability dimensions in designing the optimised diet.

## Methods

### Study population and dietary data

Observed dietary intake data among 392 healthy Japanese adults (196 men and 196 women, aged 20–69 years) living 23 out of 47 prefectures were used for the analysis in this study^(28)^. Details of the study design and participant characteristics have been reported elsewhere^(28,29)^. In brief, 400 healthy adults (200 men and 200 women) were recruited from the workers in separate welfare facilities and family members of the workers. The participants were not randomly sampled but were volunteers. With few exceptions, one person from each household participated in the survey. Recruitment was stratified by sex (men or women) and five 10-year age bands. Among the participants, 392 adults completed both the four-non-consecutive-day semi-weighed dietary records and a lifestyle questionnaire. The four recording days for the dietary records consisted of three working days and 1 d off. The survey was conducted from February to March 2013. This study was conducted according to the guidelines laid down in the Declaration of Helsinki^(30)^, and all procedures involving human subjects were approved by the Ethics Committee of the University of Tokyo, Faculty of Medicine (approval number: 10 005, approval date: 7 January 2013). The research dietitians individually explained the aims and procedure of the study to all participants. Written informed consent was obtained from all participants. Details of the procedure of dietary records have been reported elsewhere^(29)^. In brief, participants weighed ingredients in dishes including mixed dishes, prepared dishes after cooking and all drinks by using the provided equipment whenever possible. When weighing was difficult (e.g. eating out), they recorded the name of restaurant and dishes and estimated amount of leftovers. All recorded foods and beverages were assigned food item numbers according to the Standard Tables of Food Composition in Japan, Fifth Revised and Enlarged Edition^(31)^ by research dietitians. All records were checked by research dietitians both at each facility and the survey centre. Nutrient intakes except for added sugar were estimated based on the Standard Tables of Food Composition in Japan^(31)^. Added sugar intake was estimated based on a composition database recently developed for the Japanese population^(32,33)^. The average intakes during four-assessment-days were calculated.

In order to benchmark diets and calculate alternative diets in focusing on diet composition rather than weight consumed, the intake of food and nutrients was standardised to per 10·460 MJ (2500 kcal) energy intake (EI) for men and per 8·386 MJ (2000 kcal) for women. The averaged individual EI was not used to avoid complication in calculating optimised diet. Before standardisation, participants having implausible report for EI were excluded to avoid under- or over-estimation of food and nutrient intakes. If food selective misreporting occurred in implausible reporters, intakes of specific food groups or nutrients could be under- or over-estimated at standardising process of EI. Under- and over-reporting were evaluated by the ratio of EI to BMR (EI:BMR) based on the Goldberg cut-off method^(34)^. Details of this procedure have been described elsewhere^(25)^. In brief, EI:BMR was calculated by dividing average EI by BMR calculated using a sex-specific equation for the Japanese population^(35)^. Participants were identified as plausible, under- and over-reporters of EI depending on whether EI:BMR of the individual was within, below or above the 95 % confidence limits of agreement between EI:BMR and the respective physical activity level. Physical activity level for sedentary lifestyle was assumed for all subjects because there is no objective physical activity level value, and physical activity level estimated by using questionnaire regarding activities with various exercise intensities and the metabolic equivalent value for each activity was relatively low (1·55 for men and 1·57 for women on average)^(29)^. Therefore, twenty-three participants (fourteen under-reporters and nine over-reporters) were excluded from the analysis; the final sample consisted of 184 men and 185 women.

### Food group classification

Food items were reclassified according to the FBDG developed for the previous work (online Supplemental Table 1)^(24)^. The amount of legumes was calculated as cooked weight. The meat group was further disaggregated to beef, pork, other meat and chicken due to the large difference of GHGE among meat subgroups. Processed meat products were reclassified into beef or pork according to the ingredients. Dairy products were disaggregated to milk/cream/yogurt and cheese.

Whole grains were categorised based on the definition of the American Association of Cereal Chemists^(36)^. Thus, whole grains included brown rice, whole grain flour, whole barley flour, and whole grain noodle, and refined grains included well-milled rice, 70 % milled rice, half-milled rice, oats, bread, non-whole grain noodles and flour.

### Cultural acceptability

In this study, a higher similarity between modelled and observed diets was considered more culturally acceptable. For calculating modelled diets, the sum of absolute deviation of the intakes between the modelled diet and observed diet was calculated for twenty-one food groups (namely, whole grain, refined grain, potatoes, legumes, nuts and seeds, vegetables, fruits, beef, pork, other meat, chicken, fish, eggs, milk and dairy food, cheese, solid fats, oils, sugar and confectionery, alcohol beverage, sweetened beverage, and seasonings)^(22,23)^. Modelled diets with minimised the total deviation of the intakes were considered as the most culturally acceptable diets.

For interpretation purposes of modelled diets, so-called ‘diet similarity index’ was used to simply describe the similarity between modelled diets and observed diets for each individual^(22)^. Diet similarity index was defined as a ratio of the sum of the remaining amount in the modelled diets to total food consumption amount in the observed diets^(22)^. The remaining amount was estimated for each of the twenty-one food groups above as follows and then summed: when intake of food group *f* in the modelled diet was higher or equal to the observed diet, the amount of intake in the observed diet was labelled as the remaining amount. When the intake of food group *f* in the modelled diet was lower than the observed diet, the amount of intake of the modelled diet was labelled as the remaining amount. Intakes of tea/coffee and water were excluded from the calculation of diet similarity index because of its negligible contribution to nutrient intakes. Note that the remaining amount was different from the sum of the absolute deviations calculated above.

### Quality of nutrient intakes

Quality of nutrient intakes (i.e. nutritional quality) of the diets was assessed with the NRF 15.3^(20,21)^, which is calculated as the sum of the percentage of reference daily values (RDV) for fifteen qualifying nutrients minus the sum of the percentage of RDV for three disqualifying nutrients. Qualifying and disqualifying nutrients were those singled out in the dietary guidelines as being low in the diets of Americans and some subpopulations guided the choice of beneficial nutrients. ‘Disqualifying’ nutrients were those defined as such by the Food and Drug Administration and the United States Department of Agriculture (USDA)^(37)^. NRF was used because there was no measure developed to assess nutritional quality for Japanese based on scientific evidence. Although NRF was not developed for Japanese, NRF 9.3 has been examined for its applicability to Japanese^(38)^ and applied in the previous studies among Japanese^(39,40)^. Further, NRF was widely used in studies from other countries as shown in a previous review^(41)^. Here, NRF 15.3 was used instead of NRF 9.3 to take more nutrients into account in the model. Pearson’s and Spearman’s correlation coefficients of NRF 9.3 and 15.3 scores among the participants were 0·96 and 0·97, respectively. The total NRF 15.3 score was calculated with nutrient intakes per 10·460 MJ for men and per 8·386 MJ for women and expressed in percentage of a daily reference value. Each subscore for qualifying nutrients was capped at 100. Regarding disqualifying nutrients, when intake was the same or less than the reference value, a score of 0 was assigned to the subscore.

RDV were (for sex and age categories) determined based on the three types of reference values in Dietary Reference Intakes for Japanese, 2015^(35)^ (online Supplemental Table 2), namely the RDA, Adequate Intake and Tentative Dietary Goal for Preventing Lifestyle-related Diseases^(38,39)^. RDA was used for protein, vitamins A, B_12_ and C, thiamine, riboflavin, Ca, Fe, Zn and folate; Adequate Intake was used for vitamin D and E; Tentative Dietary Goal for Preventing Lifestyle-related Diseases was used for dietary fibre, K, saturated fats and Na. In terms of added sugars, the conditional recommendation advocated by the WHO on free sugar (i.e. the upper limit of 5 % of energy)^(42)^ was used following the previous studies using NRF score to Japanese^(39,40)^ because of a lack of a recommended value for added sugar in Japan. Similarly, the RDV for MUFA was determined by the report by FAO (i.e. 10–20 % of energy was recommended)^(43)^.

### Monetary cost of the diet

As a measure of dietary affordability, the monetary cost of dietary intake was estimated by linking the dietary data with retail food prices which is taken mainly from the National Retail Price Survey 2013^(44)^. The National Retail Price Survey is a national annual survey conducted by the Statistics Bureau, Ministry of Internal Affairs and Communications. The price data is collected in every month from retail stores located in 167 cities, towns and villages stratified by several factors including population size, geographical location, and industrial characteristics. In order to select representative retail stores in the area, stores were selected according to sales volume or number of employees. Annual average prices were calculated as mean values of all survey areas, weighted for population size. The linkage of the food item between Standard Tables of Food Composition in Japan 2015 and price data provided by a previous study^(45)^ was extended from 1426 to 2229 food items in Standard Tables of Food Composition in Japan 2015. Of the 2229 food and beverage items, the National Retail Price Survey provides direct matches for 1071 foods (48 %). For 1108 food items, the price values of similar foods (e.g. belonging to the same or adjacent food subgroup in Standard Tables of Food Composition in Japan) were used because there was no price value could be matched directly. For the remaining 50 food items, prices (per 100 g) from the websites of a nationally distributed supermarket (Seiyu, Japan) are used. Price data was collected from the website in 2015 not 2013 because the website in 2013 was not accessible. The consumer price index for food in 2015 was 107 when 2013 was used as reference (i.e. 100)^(46)^. Monetary cost of diets was also standardised per 10·460 MJ for men and per 8·386 MJ for women.

### Diet-related greenhouse gas emissions

Diet-related GHGE was estimated by the newly developed database using a global link input-output model in Japan^(47)^ and the Standard Tables of Food Composition in Japan^(31)^. A detailed description of data development was described elsewhere^(25)^. In brief, production-based GHGE for each food item (g CO_2_-eq/g food weight) was calculated by multiplying the production costs (i.e. unit prices of products) with producer-based GHGE intensities from the global link input-output model. Consequently, GHGE values for 354 foods and drinks for production phases were obtained. GHGE from post-production phases was not considered because GHGE of food system mainly comes from production phases^(6)^, and there is a lack of reliable life cycle assessment data including emission from post-production phase^(25)^. Then, values in 354 foods and drinks were systematically linked to 2231 food items commonly consumed among Japanese and adjusted by the wastage rate and weight change rate with the Standard Tables of Food Composition in Japan^(31)^. The detail of the procedure to assign the GHGE values was also described elsewhere^(25)^. Briefly, for 1568 (70 %) of 2231 food items, GHGE values were directly provided. For 92 food items (4 %), averaged GHGE values of some food items was assigned because more than one food of 354 foods was identified. For 546 food items (24 %), GHGE value was assigned as the value of food with comparable producing or processing process or averaged GHGE values of the food items belonging same food group. For twenty-three mixed dishes, the values were calculated as the combination of some GHGE values according to the recipe. Lastly, GHGE values for water and breast milk were assumed ‘0’. Diet-related GHGE (g CO_2_-eq/diet) was calculated by multiplying the GHGE value for food items (g CO_2_-eq/g food weight) and the mean food consumption of the four assessment days (g food weight/diet). Diet-related GHGE was also standardised 10·460 MJ for men and per 8·386 MJ for women.

### Assessment of other variables

Body height and weight were measured to the nearest 0·1 cm and 0·1 kg, respectively, with participants wearing light clothing and no shoes. These measurements, as well as blood pressure, were conducted by the research dietitians or medical staff in the welfare facilities. BMI was calculated as body weight in kilograms divided by the square of height in metres (kg/m^2^). The participants’ educational backgrounds and smoking habits were also assessed with the questionnaire.

### Calculation of optimised diets using a data envelopment analysis diet model

**Fig. 1.**
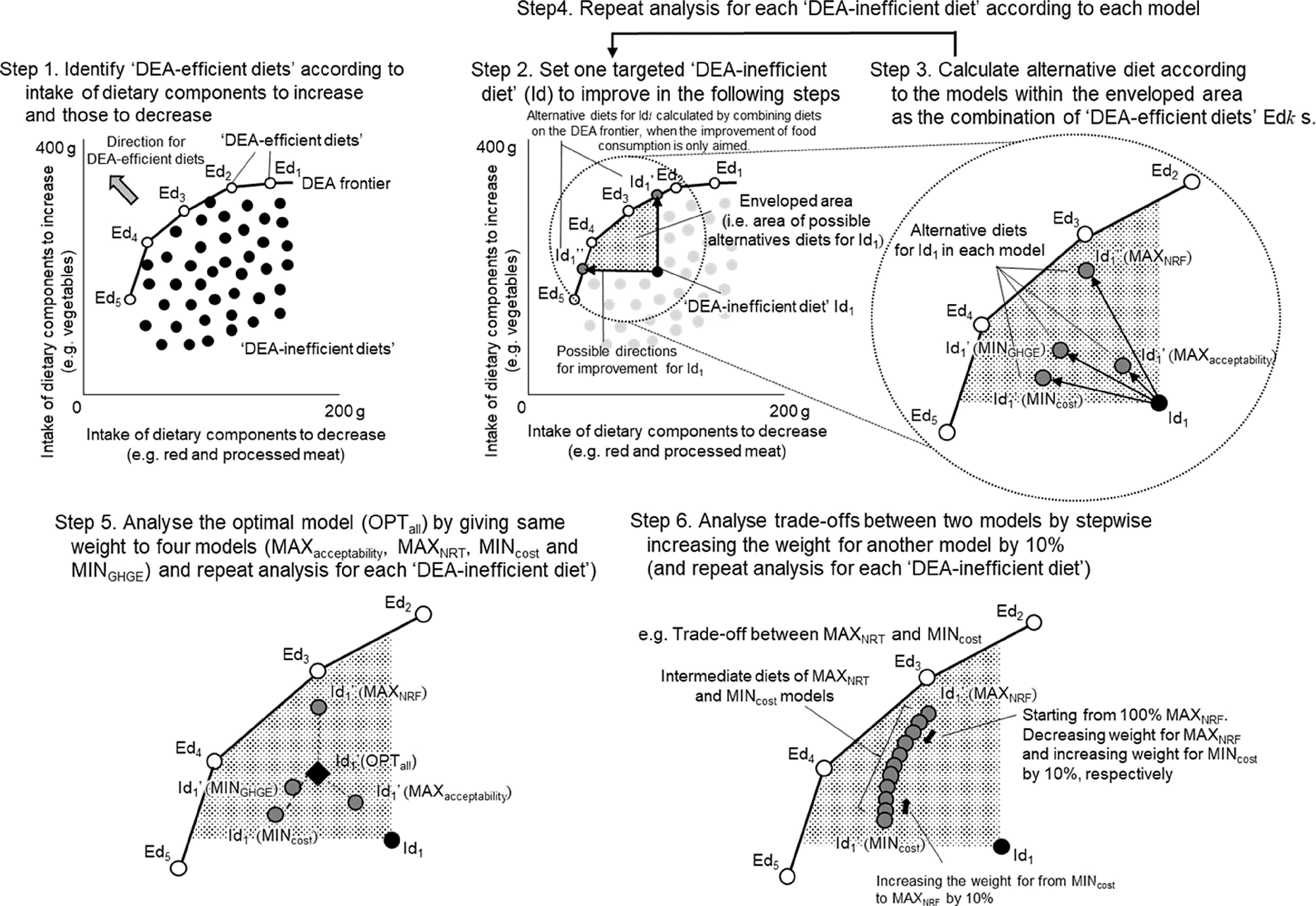
Summarize of the Data Envelopment Analysis (DEA) diet model with a two-dimensional illustrative example. Ed*k* (*k* =1, 2, 3, 4, and 5), DEA-efficient diets; Id, DEA-inefficient diet; MAX_acceptability_, the modelled diet with the most culturally acceptable (i.e. smallest change in consumption of 21 food groups from observed diets); MAX_NRF_, the modelled diet with highest Nutrient-Rich Food Index (NRF) 15.3 score assessing nutritional quality; MIN_cost_, the modelled diet with least monetary cost of diet; MIN_GHGE_, the modelled diet with the least diet-related greenhouse gas emissions; OPT_all_, the modelled diet that all selected goals (maximize cultural acceptability and NRF 15.3 score and minimise monetary cost and diet-related GHGE) were equally considered; Id_1_’ (MAX_acceptability_), Id_1_’ (MAX_NRF_), Id_1_’ (MIN_cost_), and Id_1_’ (MIN_GHGE_), Id_1_’ (OPT_all_), alternative diets for Id_1_ in MAX_acceptability_, MAX_NRF_, MIN_cost_, and MIN_GHGE_, OPT_all_ models, respectively. 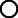, DEA-efficient diets; 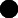, DEA-inefficient diets; 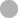, DEA-inefficient diets; 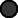, alternative diet for DEA-inefficient diets. **Step1**, DEA-efficient diets (white circles, Ed*i*) are identified by comparing the multidimensional ratio of intakes of dietary components to increase and those to decrease. Solid lines connected with white circles are the Data Envelopment Analysis (DEA) frontier. Other diets (black circles) are identified as DEA-inefficient diets. **Step 2**, for example, for a DEA-inefficient diet (black circle, Id_1_), the shaded area is a possible area of better alternatives for DEA-inefficient diet (Id_1_) because they contain lower intakes of dietary components to decrease and more intakes of dietary components to increase than the current diet Id_1_. Black arrows are two possible directions for improvement for Id_1_. Id_1_’ and Id_1_’’ (dark grey circles) are possible alternative diets when the improvement in intakes of dietary components to increase and those to decrease is only aimed. Id_1_’ and Id_1_’’ can be calculated by combining diets on the DEA frontier. **Step 3**, alternative diets for Id_1_ (dark grey circles) was calculated in each model by combining DEA-efficient diets. Black arrows are possible directions for improvement for Ed*i* when the improvement of the selected indicator is additionally considered. **Step 4**, the analysis was repeated for all DEA-inefficient diets in each model. **Step 5**, alternative diet in the OPT_all_ model for Id_1_ was calculated by weighting four models above equally. **Step 6**, trade-offs between indicators were investigated by changing weight to each model. Intermediate diets of two different models for Id_1_ (dark grey circles) are calculated by stepwise increasing weight for one model to another model by 10%. The figure shows an example of the intermediate diets between MAX_NRF_ and MIN_cost_ models.

The DEA diet model^(19)^ was used to calculate modelled diets. The analytical method is summarised in Fig. 1 with a two-dimensional illustrative example. More details of the DEA diet model used in this study are presented in the previous study^(19)^ and Appendix A-D (in the Supplemental Material) of this study. Optimised diets were calculated as a combination of existing dietary patterns expressed by food groups level but not individual food items in order to get the feasible solutions in line with FBDG at DEA.

First, observed diets were benchmarked to identify so-called ‘DEA-efficient diets’ that contain the highest (compared with all others) level of dietary components to increase for a certain level of dietary components to decrease or that contain the lowest level of dietary components to decrease for a certain level of dietary components to increase^(22)^. The rest of the diets not identified as DEA-efficient were defined as so-called ‘DEA-inefficient’ (Step1 in Fig. 1). In the benchmarking process, input- and output-oriented DEA model (Appendix A in the Supplemental Material) was used to calculate multidimensional ratio according to Banker, Charnes and Cooper models^(48)^. In this process, the efficiency score 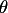 was calculated for each diet. As a result of the analysis, the diet with 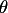 = 1 was identified as DEA-efficient (Appendix A in the Supplemental Material). The dietary components to increase and those to the decrease were selected from the previously defined FBDG^(22,24)^. The dietary components to increase included fruits, vegetables, legumes, nuts/seeds, milk/cream/yogurt, fish/seafood and whole grains. The dietary components to decrease included red and processed meat, refined grains, sweetened beverages and ethanol (as a proxy of alcoholic beverage). In addition, vitamin A (as a nutrient to increase), Na and added sugar (as nutrients to decrease) were included to be safeguarded, that is, to avoid unwanted decrease or increase intakes of these nutrients in modelled diets. Zero intakes for dietary components to increase and those to the decrease were replaced by non-zero values, that is, the lowest non-zero intake among the participants divided by 2. The proportion of zero intakes were 0 % for vegetables, red meat, refined grains, vitamin A, Na, and added sugar for both men and women, less than 10 % for fruit, legume, fish, and ethanol, 16 % (both men and women) for nuts, 31 % (men) and 33 % (women) for whole grain, and 56 % (both men and women) for sweetened beverages. This replacement was applied only in the benchmarking analysis to avoid zero values in denominators of the multidimensional ratio of intake.

Next, for the participants with DEA-inefficient diets, the alternative diets were calculated as linear combinations of observed DEA-efficient diets (Step 2 in Fig. 1). By changing the proportion of each DEA-inefficient diet ( 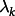 ) in the combinations, modelled diets that optimised a certain indicator were obtained (Step 2 and 3 in Fig. 1). The proportion of each DEA-efficient diet in the combinations was obtained by solving the linear programming (Appendix B and C) for three types of models: maximum/minimum models (Step 4 in Fig. 1), optimal model (Step 5 in Fig. 1) and trade-off models (Step 6 in Fig. 1). Minimum/maximum models aimed to obtain diets achieving one of four goals, separately: (1) maximum cultural acceptability (MAX_acceptability_), (2) maximum NRF 15.3 score (MAX_NRF_), (3) minimum monetary cost (MIN_cost_) and (4) minimum diet-related GHGE (MIN_GHGE_). There were three constraints to increase (or decrease) consumption of ‘the dietary components to increase (or decrease)’ and that to keep EI at the same amount of the current diets. The optimal model considered all four goals in one model simultaneously (OPT_all_). Trade-off models were aimed to examine the trade-offs between the goals, especially MAX_NRF_, MIN_cost_ and MIN_GHGE_.

To compose the linear programming model, firstly, four decision variables were formulated (Appendix C). In maximum/minimum models, full weight was assigned for the targeted goal and zero weight was assigned for the rest (Appendix D). In the OPT_all_ model, same weights were assigned for all goals. In trade-off models, nine intermediate modelled diets between MAX_NRF_ *v.* MIN_cost_, MAX_NRF_ *v.* MIN_GHGE_ and MIN_cost_ *v.* MIN_GHGE_ were calculated by applying the stepwise change of the weights by 10 %, respectively.

### Statistical analysis

SAS statistical software version 9.4 (SAS Institutee Inc., Cary, NC, USA) was used to merge and arrange dietary data and conduct statistical analysis. After the dietary data were transferred to Excel files, FICO^®^ Xpress-IVE version 1.25 was used to identify DEA-efficient diets by solving the DEA diet model. Basic characteristics and observed intakes of food and nutrients between the participants with DEA-efficient diets and those with DEA-inefficient diets were compared using the *t* test and *χ* ^2^ test with SAS. Other nutrients were compared between the participants in addition to those included in NRF in order to compare the nutrient profile among the participants in detail. All reported *P*-values are two-tailed, and *P* < 0·05 was considered significant. Cohen’s d was calculated as the difference between the means divided by the pooled standard deviations. Cramér’s V was calculated for categorical variables as a measure of association between two nominal variables. Cramér’s V goes from 0 to 1, where 1 indicates strong association. Dietary intakes, NRF 15.3 score, diet-related GHGE, monetary cost and diet similarity index in the modelled diets were compared with those in observed diet using paired *t* tests. *P*-values were corrected for multiple comparisons by using the Benjamini–Hochberg approach^(49)^.

Following a previous study^(12)^, we assumed that dietary modification was required when the difference in intakes of each food group between the observed and modelled diets was more than +10% or −10 %^(12)^ on average and the difference was statistically significant. This assumption was made for convenience in data interpretation.

## Results

Participants identified as having DEA-efficient diets had no significant difference in demographic variables from those with DEA-inefficient diets (Table 1), while they had a higher NRF 15.3 score (men), diet-related GHGE (women) and dietary cost (women). Moreover, participants with DEA-efficient diets had higher intakes of dietary components to increase, protein, dietary fibre, vitamins, and other micronutrients and lower intakes of dietary components to decrease (Table 2 and online Supplemental Tables 3 and 4). Results of detailed analysis for comparing food and nutrient intakes between participants with DEA-efficient diets and those with DEA-inefficient diets were described in the Supplemental Material.

**Table 1.** Basic characteristics of all participants, participants with DEA-efficient diets and those with DEA-inefficient diets among 184 Japanese men and 185 women

	Men									Women								
	All (*n* 184)		With DEA-efficient diets* (*n* 74)		With DEA-inefficient diets* (*n* 110)					All (*n* 185)		With DEA-efficient diets* (*n* 71)		With DEA-inefficient diets* (*n* 114)				
	Mean or *n*	sd or (%)	Mean or *n*	sd or (%)	Mean or *n*	sd or (%)	Cohen’s d†	Cramér’s V‡	*P* §	Mean or *n*	sd or (%)	Mean or *n*	sd or (%)	Mean or *n*	sd or (%)	Cohen’s d†	Cramér’s V‡	*P* §
Age (years)	45·0	13·1	45·4	13·8	44·8	12·7	0·046		0·43	44·4	13·4	46·8	13·3	43·0	13·3	0·28		1·00
BMI (kg/m^2^)	23·9	3·4	23·8	3·4	24·0	3·4	0·059		0·86	22·5	3·4	22·8	3·4	22·3	3·3	0·15		0·74
Height (cm)	170·3	5·4	169·6	5·3	170·7	5·5	0·20		0·80	157·4	5·7	157·0	5·8	157·7	5·7	0·12		0·84
Weight (kg)	69·4	11·1	68·5	11·7	70·0	10·7	0·14		0·39	55·7	8·8	56·4	9·3	55·3	8·5	0·13		0·38
Education level (*n*, %)																		
Junior high school	4	2	0	0	4	4		0·17	0·15	6	3	3	4	3	3		0·048	0·93
Senior high school	34	18	14	19	20	18				61	33	24	32	37	37			
Two-year college/professional training college	50	27	16	21	34	31				83	45	31	42	52	51			
University/graduate school	96	52	44	59	52	48				35	19	13	18	22	22			
Smoking habit (*n*, %)																		
Non-smoker	62	34	31	41	31	28		0·14	0·15	145	78	59	80	86	85		0·10	0·39
Past smoker	54	29	20	27	34	31				13	7	3	4	10	10			
Smoker	68	37	23	31	45	41				27	15	9	12	18	18			
	(per 10·460 MJ)						(per 8·368 MJ)											
Nutrient-Rich Food Index 15.3 score	1226	128	1243	147	1214	112	0·23		0·01	1222	121	1249	123	1203	116	0·38		0·53
Dietary cost (Japanese yen)	1251	215	1261	224	1245	209	0·074		0·50	1061	199	1119	245	1022	149	0·49		<0.0001
Diet-related greenhouse gas emissions (g CO_2_eq)	4668	997	4627	952	4696	1031	0·069		0·47	3946	886	4011	1071	3903	740	0·12		0·0004

DEA, data envelopment analysis.

* ‘DEA-efficient diets’ were identified as the diets having a higher multidimensional ratio of predefined ‘dietary components to increase’ per unit of ‘dietary components to decrease’ by using data envelopment analysis. The rest of diets were defined as ‘DEA-inefficient diets’.

† Cohen’s d, an effect size used to indicate the standardised difference between two means, was calculated as the difference between the means divided by the pooled sd.

‡ Cramér’s V is a measure of association between two nominal variables. It goes from 0 to 1, where 1 indicates strong association.

§ The *t* test was performed for continuous variables; and the χ^2^ test, for categorical variables. *P* < 0·05 was considered statistically significant.

**Table 2.** Comparison of intake of food and nutrients (per 10·460 MJ for men and per 8·386 MJ for women) used to benchmark diets in observed diet between participants with DEA-efficient diets and those with DEA-inefficient diets, 184 japanese men and 185 women*

	Men						Women					
	With DEA-efficient diets* (*n* 74)		With DEA-inefficient diets* (*n* 110)				With DEA-efficient diets (*n* 71)		With DEA-inefficient diets* (*n* 114)			
	Mean	sd	Mean	sd	Cohen’s d†	*P* ‡	Mean	sd	Mean	sd	Cohen’s d†	*P* ‡
Whole grain (g)§	10	57	2	11	0·20	< 0·0001	5	31	1	4	0·21	< 0·0001
Refined grain (g)||	532	167	579	107	0·35	< 0·0001	390	112	409	76	0·20	0·0003
Legumes (g)§	73	73	51	42	0·39	< 0·0001	72	59	44	30	0·62	< 0·0001
Nuts (g)§	6	14	3	5	0·36	< 0·0001	4	7	3	4	0·19	< 0·0001
Vegetables (g)§	309	146	274	92	0·30	< 0·0001	321	159	263	94	0·46	< 0·0001
Fruits (g)§	121	135	65	64	0·55	< 0·0001	118	113	86	77	0·34	0·0003
Red and processed meat (g)||	74	49	81	39	0·16	0·03	42	33	59	27	0·57	0·04
Fish (g)§	85	57	79	42	0·13	0·003	76	52	61	34	0·36	< 0·0001
Milk and other dairy products (g)§	114	123	74	74	0·40	< 0·0001	125	116	99	79	0·27	0·0002
Alcoholic beverages (g)||	196	328	234	317	0·12	0·73	70	169	64	151	0·036	0·28
Sweetened beverages (g)||	42	127	51	89	0·091	0·001	28	59	40	78	0·16	0·01
Na (mg)||	4554	1128	4746	854	0·20	0·01	3848	1159	3876	737	0·03	< 0·0001
Vitamin A (µg RAE)§	956	1370	498	192	0·51	< 0·0001	667	734	462	154	0·43	0·01
Alcohol (g)||,¶	18·7	31·3	17·0	21·0	0·065	0·0002	5·2	10·2	4·0	7·6	0·14	0·005
Added sugar (g)||	34·6	24·5	37·9	17·8	0·16	0·002	32·5	15·8	38·6	14·9	0·39	0·59

DEA, data envelopment analysis.

* ‘DEA-efficient diets’ were identified as the diets having a higher multidimensional ratio of predefined ‘dietary components to increase’ per unit of ‘dietary components to decrease’ by using data envelopment analysis. The rest of diets were defined as ‘DEA-inefficient diets’.

† Cohen’s d, an effect size used to indicate the standardised difference between two means, was calculated as the difference between the means divided by the pooled sd.

‡ The *t* test was performed. *P* < 0·05 was considered statistically significant.

§ Food group included as ‘dietary components to increase’ in DEA.

|| Food group included as ‘dietary components to decrease’ in DEA.

¶ Used as a proxy for alcoholic beverages in benchmarking diets.

Food consumption in observed diets and modelled diets were shown in Table 3. Compared with the observed diets, all four minimum/maximum models and the optimal model considering all goals provided larger consumption of whole grains, legumes, nuts/seeds, and fruits, and lower consumption of alcoholic and sweetened beverages among both men and women.

**Table 3.** Food intake (g/10·460 MJ for men and per 8·386 MJ for women) in observed diets and modelled diets among 184 Japanese men and 185 women*

	Men (*n* 184)												Women (*n* 185)											
	Observed		MAX_acceptability_		MAX_NRF_		MIN_cost_		MIN_GHGE_		OPT_all_		Observed		MAX_acceptability_		MAX_NRF_		MIN_cost_		MIN_GHGE_		OPT_all_	
	Mean	sd	Mean	sd	Mean	sd	Mean	sd	Mean	sd	Mean	sd	Mean	sd	Mean	sd	Mean	sd	Mean	sd	Mean	sd	Mean	sd
Cereals	565	133	562	125	532	109^a^	571	125	598	133^a^	582	126^a^	404	89	400	84^a^	379	81^a^	402	82	401	82	403	81
Whole grains†	5	38	**12**	**4**1^a^	**37**	**53** ^**a**^	**21**	**48** ^**a**^	**53**	**71** ^**a**^	**31**	**54** ^**a**^	2	19	**4**	**20** ^**a**^	**4**	**19** ^**a**^	**10**	**24** ^**a**^	**12**	**26** ^**a**^	**9**	**24** ^**a**^
Refined grains‡	560	136	550	131^a^	**495**	**120** ^**a**^	550	133^a^	545	132^a^	551	133^a^	402	92	396	88^a^	375	84^a^	393	87^a^	388	87^a^	393	87^a^
Potatoes	52	39	53	28	56	28	57	29	52	30	55	29	42	35	41	23	35	23	38	25	37	23	38	23
Legumes and nuts	64	57	**77**	**56** ^**a**^	**97**	**60** ^**a**^	**87**	**54** ^**a**^	**87**	**55** ^**a**^	**85**	**54** ^**a**^	58	47	**66**	**44** ^**a**^	**81**	**41** ^**a**^	**77**	**43** ^**a**^	**80**	**45** ^**a**^	**74**	**42** ^**a**^
Legumes†	60	57	**71**	**56** ^**a**^	**92**	**61** ^**a**^	**82**	**55** ^**a**^	**81**	**56** ^**a**^	**79**	**55** ^**a**^	55	46	**62**	**43** ^**a**^	**73**	**40** ^**a**^	**72**	**42** ^**a**^	**72**	**44** ^**a**^	**68**	**41** ^**a**^
Nuts/seeds†	4·2	9·6	**5·4**	**9·6** ^**a**^	**5·3**	**9·5** ^**a**^	**5·4**	**9·9** ^**a**^	**5·4**	**9·6** ^**a**^	**5·5**	**9·6** ^**a**^	3·4	5·6	**4·3**	**5·5** ^**a**^	**8·2**	**6·6** ^**a**^	**5·0**	**5·6** ^**a**^	**7·8**	**7·8** ^**a**^	**6·4**	**6·4** ^**a**^
Vegetables†	288	117	308	108^a^	**366**	**110** ^**a**^	311	108^a^	**334**	**108** ^**a**^	316	105^a^	285	126	293	120^a^	**371**	**116** ^**a**^	294	119^a^	299	116^a^	299	115^a^
Fruits†	87	102	**106**	**97** ^**a**^	**133**	**91** ^**a**^	**108**	**99** ^**a**^	**121**	**94** ^**a**^	**113**	**95** ^**a**^	98	94	**115**	**90** ^**a**^	**225**	**126** ^**a**^	**124**	**88** ^**a**^	**154**	**91** ^**a**^	**126**	**87** ^**a**^
Fish/seafood†	82	49	89	45^a^	**96**	**43** ^**a**^	87	45^a^	**91**	**45** ^**a**^	87	45^a^	67	42	74	39^a^	**78**	**38** ^**a**^	71	40^a^	73	39^a^	72	39^a^
Meat	116	56	110	47^**a**^	**122**	**46** ^**a**^	111	49	99	46^a^	107	46^a^	81	43	**68**	**36** ^**a**^	76	36^a^	**56**	**35** ^**a**^	**59**	**37** ^**a**^	**62**	**34** ^**a**^
Red and processed meat‡	78	43	**70**	**37** ^**a**^	71	37^a^	**59**	**36** ^**a**^	**53**	**37** ^**a**^	**59**	**36** ^**a**^	52	31	**41**	**24** ^**a**^	**39**	**22** ^**a**^	**34**	**23** ^**a**^	**35**	**24** ^**a**^	**37**	**22** ^**a**^
Beef	22	24	**18**	**18** ^**a**^	21	19	**14**	**17** ^**a**^	**11**	**18** ^**a**^	**14**	**17** ^**a**^	13	17	**10**	**12** ^**a**^	**16**	**13**	**7**	**12** ^**a**^	**6**	**12** ^**a**^	**6**	**12** ^**a**^
Pork	57	35	52	28^a^	**50**	**26** ^**a**^	**45**	**26** ^**a**^	**42**	**26** ^**a**^	**45**	**26** ^**a**^	39	28	**31**	**21** ^**a**^	**23**	**20** ^**a**^	**27**	**20** ^**a**^	**30**	**22** ^**a**^	**31**	**20** ^**a**^
Other meat	0·09	0·58	0·03	0·23	0·02	0·22	0·04	0·23	0·03	0·22	0·09	0·58	0·03	0·22	0·26	1·93	0·05	0·32	0·03	0·31	0·03	0·31	0·03	0·31
Chicken	37	33	40	26	**51**	**27** ^**a**^	**52**	**32** ^**a**^	**47**	**27** ^**a**^	**48**	**28** ^**a**^	28	28	28	22	**37**	**24** ^**a**^	**21**	**21** ^**a**^	**24**	**22** ^**a**^	**25**	**21** ^**a**^
Egg	45	23	41	18^a^	**36**	**18** ^**a**^	41	20^a^	**37**	**19** ^**a**^	41	19^a^	38	23	**43**	**18** ^**a**^	**53**	**21** ^**a**^	**45**	**18** ^**a**^	39	18	**44**	**17** ^**a**^
Dairy products	94	98	**114**	**91** ^**a**^	**191**	**112** ^**a**^	**142**	**95** ^**a**^	**145**	**98** ^**a**^	**130**	**92** ^**a**^	113	96	122	90^a^	**164**	**86** ^**a**^	**140**	**90** ^**a**^	**139**	**86** ^**a**^	**134**	**86** ^**a**^
Milk/cream/yogurt†	90	98	**109**	**92** ^**a**^	**187**	**113** ^**a**^	**138**	**94** ^**a**^	**142**	**98** ^**a**^	**126**	**92** ^**a**^	109	96	118	90^a^	**160**	**86** ^**a**^	**135**	**90** ^**a**^	**134**	**86** ^**a**^	**128**	**87** ^**a**^
Cheese	4·1	6·2	5·0	5·4	3·8	4·9	4·0	5·4	3·7	5·1	3·9	5·1	3·7	5·8	4·0	5·0	3·9	4·8	**5·2**	**5·5** ^**a**^	**5·3**	**5·7** ^**a**^	**5·7**	**5·4** ^**a**^
Fats and oils	24	9	23	7	**22**	**8** ^**a**^	**22**	**8** ^**a**^	**23**	**8** ^**a**^	23	8	20	9	22	9	**17**	**6** ^**a**^	**22**	**9** ^**a**^	21	8	**22**	**9** ^**a**^
Solid fats	2·6	3·3	2·5	2·1	2·2	2·0	2·6	2·2	**2·1**	**2·1** ^**a**^	2·3	2·1	2·7	3·6	**3·4**	**3·1** ^**a**^	1·3	2·2	3·2	3·2	3·0	2·8	3·0	3·0
Oils	21	9	21	7	20	7^a^	20	8	20	7	21	7	18	8	18	7	15	5	19	7	18	6	19	7
Sugar/confectioneries	53	39	54	34	**46**	**31** ^**a**^	52	37	**48**	**35** ^**a**^	**47**	**32** ^**a**^	64	36	62	29	**55**	**27** ^**a**^	59	29^a^	**53**	**29** ^**a**^	**57**	**28** ^**a**^
Alcoholic beverages‡	219	321	**185**	**264** ^**a**^	**131**	**234** ^**a**^	**125**	**231** ^**a**^	**121**	**237** ^**a**^	**147**	**239** ^**a**^	66	158	**58**	**135** ^**a**^	**37**	**112** ^**a**^	**30**	**109** ^**a**^	**32**	**110** ^**a**^	**40**	**114** ^**a**^
Tea/coffee	643	427	652	323	686	314	600	314	633	312	600	312	655	391	651	284	791	290	624	288	616	300	641	284
Sweetened beverages‡	47	106	**32**	**90^a^**	**23**	**83** ^**a**^	**22**	**85** ^**a**^	**21**	**84** ^**a**^	**25**	**85** ^**a**^	35	72	**20**	**43** ^**a**^	**12**	**39** ^**a**^	**17**	**41** ^**a**^	**14**	**41** ^**a**^	**15**	**39** ^**a**^
Seasonings	142	103	132	75^a^	**111**	**69** ^**a**^	**116**	**70** ^**a**^	**102**	**70** ^**a**^	**112**	**68** ^**a**^	115	76	**99**	**52** ^**a**^	**80**	**52** ^**a**^	**96**	**52** ^**a**^	**89**	**51** ^**a**^	**89**	**51** ^**a**^
Water	548	389	578	343	592	329	**616**	**342** ^**a**^	576	327	592	330	533	331	534	262	622	261	515	252	512	250	511	249

MAX_acceptability_, the modelled diets with the most culturally acceptable (i.e. smallest change in food groups consumption from observed diets); MAX_NRF_, the modelled diets with the highest Nutrient-Rich Food Index (NRF) 15.3 score assessing nutritional quality; MIN_cost_, the modelled diets with least monetary cost of diet; MIN_GHGE_, the modelled diet with the least diet-related greenhouse gas emissions; OPT_all_, the modelled diet that all four goals were equally considered.

^a^Values with superscript letters within a row are significantly different from the values in observed diets by paired *t* tests. Statistical significance was determined by a corrected two-sided *P* < 0·05 by the Benjamini–Hochberg approach^(49)^ method.

* Values are mean and sd of nutrient intake in the observed diets and modelled diets. Values are in bold when food intakes in the modelled diet are more than 10% or –10 % differ on average from those in observed diets and differences are statistically significant.

† Food group included in data envelopment analysis as dietary components to increase.

‡ Food group included in data envelopment analysis as dietary components to decrease.

**Fig. 2.**
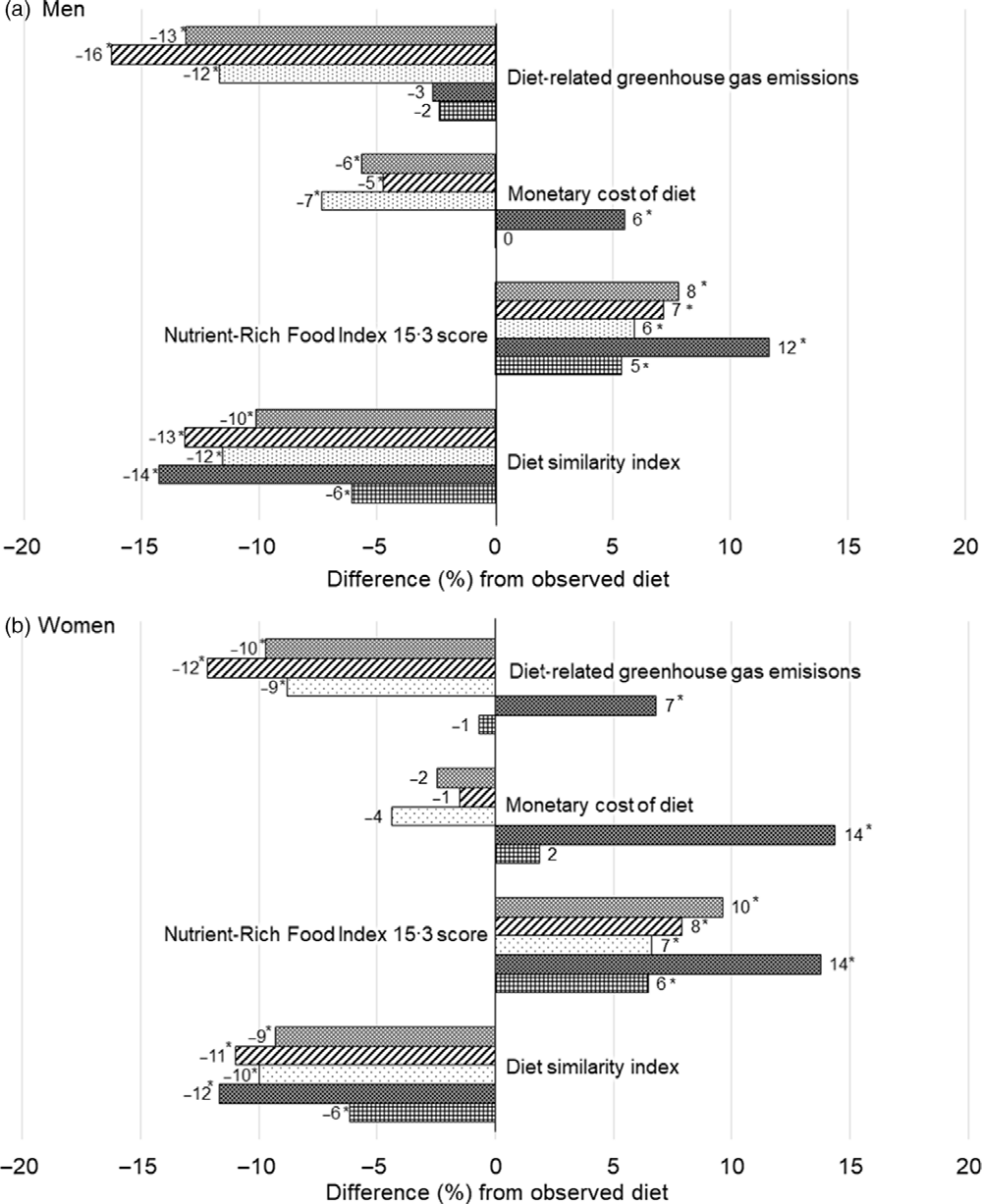
The proportion of the difference in diet similarity index, Nutrient-Rich Food Index 15.3 score, monetary cost of diet and diet-related greenhouse gas emissions in modelled diets compared with observed diets among (a) 184 men and (b) 185 women. *Significantly differed from the observed diet, tested by paired *t* test. *P* < 0·05 was considered as statistically significant with the correction for multiple comparisons by using the Benjamini–Hochberg approach^(49)^ considering multiple measurements, model to maximise cultural acceptability (MAX_accecptability_), model to maximise NRF 15.3 score (MAX_NRF_), model to minimise monetary diet cost (MIN_cost_), model to minimise diet-related GHGE (MIN_GHGE_), and the optimal model considering all four goals (OPT_all_). GHGE, greenhouse gas emissions.

In the MAX_acceptability_ models to maximise cultural acceptability, demanded changes in food consumption were generally small (namely, on average less than +30 % or –30 %) except for whole grains and sweetened beverages compared with those in the other four models. In addition to the food groups listed above, consumption was increased for milk/cream/yogurt (in men only) and eggs (in women only) and decreased in beef, pork, and seasonings (women). The decrease in similarity index was smallest (by 6 % for men and women) than other models (7 %–16 % reduction), whereas NRF 15.3 score was a bit increased (by 5 % for men and 6 % for women) (Fig. 2). No significant difference was shown for monetary diet cost and diet-related GHGE. In the MAX_NRF_ model that maximised the NRF 15.3 score, consumption was increased for whole grains, legumes, nuts/seeds, vegetables, fruits, fish/seafood, beef (women), chicken, eggs (women) and milk/cream/yogurt compared with observed diets. Consumption was decreased for refined grains (men), potatoes (women), pork, and eggs (men), and seasonings as well as alcoholic and sweetened beverages. NRF 15.3 score was increased (by 12 % for men and 14 % for women); however, the monetary cost in men (by 6 %) and women (by 14 %) and diet-related GHGE in women (by 7 %) were also increased. In the MIN_cost_ diet that minimised the monetary cost of diet, consumption was increased in chicken (men) and milk/cream/yogurt, and cheese (women) in addition to whole grains, legumes, nuts/seeds, and fruits. The decreased consumption was shown in beef, pork, chicken (women), alcoholic and sweetened beverages, and seasonings. The monetary cost was decreased by 7 % for men and 4 % for women. Additionally, diet-related GHGE was decreased (by 12 % for men and 9 % for women) and NRF 15.3 score was increased (by 6 % for men and 5 % for women). In MIN_GHGE_ diet to minimise diet-related GHGE, although its food intake pattern was generally similar to those in MIN_cost_ diets, it was characterised by a large increase in whole grains intake and decrease in sugar/confectioneries intake. In men, an increase in vegetables and decrease in eggs and solid fats was additionally demanded. Diet-related GHGE was decreased by 16 % for men and 12 % for women. In addition, the monetary cost was decreased in men (5 %) and NRF 15.3 scores increased for both men (7 %) and women (8 %).

In the optimal model (OPT_all_) considering all goals, demanded changes in food consumption were similar to those in MIN_cost_ and MIN_GHGE_. Food consumption was increased for whole grains, legumes, nuts/seeds, fruits, chicken (men), eggs (women), milk/cream/yogurt and cheese (women). Consumption was decreased for beef, pork, sugar/confectioneries, alcoholic and sweetened beverages, and seasonings. NRF 15.3 score increased (8 % for men and 10 % for women) and diet-related GHGE decreased (13 % for men and 10 % for women), whereas monetary cost decreased in men (6 %) but did not significantly change in women.

Nutrient intakes in observed diets and modelled diets were compared in Supplemental Table 5. Intakes for macronutrients in the modelled diets were similar levels to the observed diets irrespective of the models. In contrast, intakes of dietary fibre, vitamins, K, and Mg were increased and intakes of Na, added sugar, and alcohol were decreased in all models compared with observed diets.

**Fig. 3.**
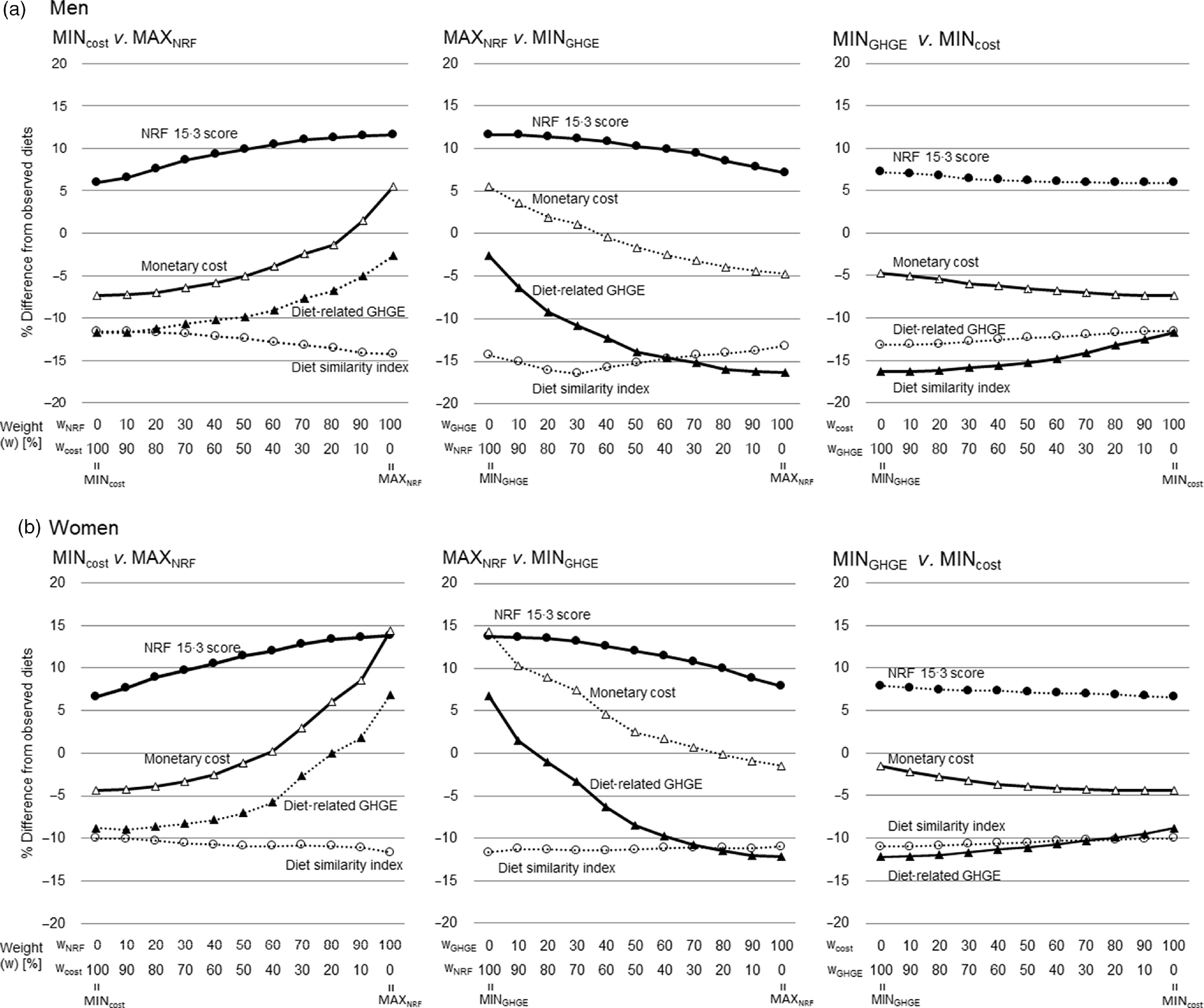
The proportion of the difference in dietary diet similarity index, Nutrient-Rich Food Index (NRF) 15.3 score, monetary cost of diet and diet-related greenhouse gas emissions from observed diet and modelled diet in trade-off analysis for (a) 184 men and (b) 185 women. MAX_NRF_, the modelled diet with the highest nutritional quality assessed by NRF 15.3 score; MIN_cost_, the modelled diet with least monetary cost of the diet; MIN_GHGE_, the modelled diet with the least diet-related greenhouse gas emissions (GHGE). Reference is the observed diet (0 % in vertical lines) and the vertical line shows the weight of the two models examined trade-offs between MIN_cost_ *v.* MAX_NRF_ (left), MAX_NRF_ *v.* MIN_GHGE_ (middle), MIN_GHGE_ *v.* MIN_cost_ (right). ○, diet similarity index; ●, NRF 15.3 score; Δ, monetary cost; ▲, diet-related GHGE. Solid lines represent two target variables whose weight was changed to examine trade-off. Dotted lines represent other two variables whose weight was not changed.

Trade-offs were found between NRF 15.3 score *v.* diet-related GHGE, NRF 15.3 score *v.* monetary cost and diet-related GHGE *v.* monetary cost (Fig. 3). When the weight for MAX_NRF_ was increased while the weight for the MIN_cost_ or MIN_GHGE_ was decreased, the NRF 15.3 score gradually improved but the monetary cost or diet-related GHGE was also increased unwantedly. When the weight for the MIN_cost_ was increased and weight for MIN_GHGE_ was decreased, diet-related GHGE was gradually decreased and the monetary cost was increased.

## Discussion

This is the first study to design alternative diets based on a DEA diet model considering cultural acceptability, nutritional quality, the monetary cost of diets and diet-related GHGE among Japanese. More sustainable and acceptable dietary patterns were shown by the optimal model (OPT_all_) considering all four goals, but improvement would be at a moderate level due to trade-offs between the indicators. Trade-offs between the selected indicators were explicitly shown, especially between nutritional quality and monetary cost or diet-related GHGE. Dietary intake patterns of the optimal model (OPT_all_) demanded increases in consumption of whole grains, legumes, nuts/seeds, fruits, and milk/cream/yogurt, and decreases in consumption of red and processed meat, sugar/confectioneries, alcoholic and sweetened beverages, and seasonings. These changes in food consumption would be the first step towards a more sustainable and cultural acceptable diet for Japanese. However, the generalisability of the results was low due to the small and non-representative sample of this study. Thus, the results of this study should be interpreted as experimental material to test the procedure to model sustainable diets with a representative and random selected sample.

The trade-offs between nutritional quality and monetary cost or diet-related GHGE were consistently shown in the previous modelling and descriptive studies^(14,17,18,26,27)^, whereas optimising for the achievement of higher nutritional quality was not necessarily associated with reduced GHGE^(14,17,18,26)^. In addition, acceptable and healthier diets were associated with higher dietary costs^(27)^. These results suggest that dietary changes that only aimed to improve nutritional quality could not result in sustainable diets from the perspective of climatic impact and affordability. On the other hand, there were correlations between monetary cost and diet-related GHGE in this study. This might be due to the similarity of major food contributors to monetary cost^(45)^ and diet-related GHGE^(25)^ among Japanese. Food with higher monetary costs such as meat and fish also have higher GHGE in the production phase. Thus, these similarities of major food contributors would result in a similar food consumption pattern and proportion of improvement in the modelled diet minimising monetary diet cost and that minimising diet-related GHGE.

With regard to the food intake patterns, all four maximum/minimum models and the weighed optimal model demanded an increased consumption of whole grains, legumes, and fruits and decreased consumption of alcoholic and sweetened beverages compared with the observed diets. Demanded changes in consumption of these food groups from the observed diets were consistent with several previous studies^(13,14,17)^. Thus, dietary changes for these food groups would be widely needed to achieve sustainable diets. Similar to previous studies in Western countries, consumption of red and processed meat (including beef and pork) was also decreased in the modelled diet minimising monetary diet cost and that minimising diet-related GHGE, and the diet with optimal models. However, demanded decreases in red and processed meat were relatively small (from −35 % to −11 %) and beef intake was increased in MAX_NRF_ model for women, while much larger reductions were demanded in Western diets. This might be explained by the relatively low meat intake among Japanese. Thus, a drastic reduction of red and meat processed products might not have a large benefit for the Japanese population when nutritional quality was considered.

A previous study from Japan used a linear programming model to focus on the adequacy of nutrient intakes^(12)^. Our result for the MAX_NRF_ model was consistent with that previous study achieving large improvements for intakes of protein, dietary fibre, vitamins and K compared with the observed diet. Large improvements in these nutrients would be associated with increasing meat intake as well as vegetables and other protein-rich foods. Demanding an increase in meat intake was also shown in the previous study^(12)^. These results suggest that increasing meat consumption have some benefit for nutrition intake among Japanese, because current meat consumption among Japanese is relatively small compared with Western countries and meat is a major source of protein, vitamins and K. On the other hand, intakes of total fat and saturated fat in the modelled diets were similar to the observed diet in this study, while they were decreased in the previous study^(12)^. This can be explained by small differences in the intake of total fat and saturated fat intakes between participants with DEA-efficient diets and the other participants (online Supplemental Table 3), whereas the linear programming model in the other study^(12)^ allowed to force fat intake beyond the range of food intakes in our study population. In addition, Na intake in the MAX_NRF_ diet was slightly lower than in the observed diets but still above the recommended value, while in the previous study it was lowered to the reference values^(12)^. This suggests that achieving the nutritional goal of saturated fat and Na might be difficult by dietary modifications within the range of current dietary habits and that food reformulation by food companies would be necessary.

In the present study, adequacy of nutrient intake was not fully achieved for some reasons, although fully achievement was not the aim of DEA diet model. First, there were only small or no differences in nutrient intakes between participants with DEA-efficient diets and those with DEA-inefficient diets. There would be some similarities in the dietary habits among participants working at welfare facilities. Thus, the proportion of participants identified as DEA-efficient diets resulted in relatively high (i.e. around 40 %). Second, DEA-efficient diets did not have desirable amounts for all dietary components to increase and those to decrease; this is because the DEA diet model benchmarks diets within the observed diets where the fully desirable diet was rarely observed. Third, improvement of the nutritional quality could have been limited by using NRF 15.3 including only eighteen nutrients. However, improvement of nutrient intakes would be small irrespective of the measures for nutritional quality due to these small or no difference between the participants. Moreover, there is no other applicable measure to assess the quality of nutrient intakes for Japanese other than NRF. Thus, further research with a larger sample size and larger variation of the diet in the sample would be needed to obtain alternative diets with large improvement. Furthermore, further research is also needed to examine the actual feasibility of the optimised diet with regard to the trade-offs between feasibility of the optimised diet and achievement of the adequacy of nutrient intake.

Regarding diet-related GHGE, we had to use a different database and system boundaries for GHGE as other studies, which limits comparability to other studies. We observed that the percentage reduction of diet-related GHGE in the modelled diet was at most 16 %, that is, smaller as in most other studies, where more than 25 % reduction was calculated in the optimised diet by linear programming^(13,14,17,50)^. Similarly to nutrient intakes, the improvement of diet-related GHGE might be limited due to a small difference of diet-related GHGE between participants and a relatively large proportion of participants identified as DEA-efficient diets. This illustrates the difficulty of modelling diets within the boundary of the observed diets to achieve a large reduction of diet-related GHGE or a large increase of nutritional quality. However, the objective of the benchmarking approach was not to find a dietary pattern with the largest possible improvement of indicators by a drastic dietary change but to find a feasible dietary pattern with some improvement within the observed range of diets. Thus, moderate improvements obtained in this study were reasonable for the study aim. Introducing additional Japanese population groups would allow a wider set of solutions for modelled diets with larger gains health and sustainability, but still within the context of the Japanese diets.

There are several strengths in this study. We used a newly developed method with a benchmarking approach, which aims to propose alternative diets within the area of observed diets by combining existing dietary intake patterns. This approach could be used as a complementary manner for a mathematical optimisation model. The strength of the DEA diet model is higher feasibility of the modelled diets because it is calculated by combining the DEA-efficient diets, which are existing whole diets and actually consumed by some of the participants. The DEA diet model provides a proportion of each existing DEA-efficient diet composing modelled diets in addition to the summed amount of intake of each food item or group per meal, day, or year in the modelled diet. The existing DEA-efficient diet composing modelled diets can be specific examples of the dietary pattern. However, caution is needed when applying the DEA diet model because this model limits the degree of change from the observed diet due to its methodological characteristics. Thus, this method should be used when the study aims to obtain an optimised diet with a particular emphasis on feasibility. In addition, further study would be needed to truly proof of the concept of the DEA-diet model because the advantage of the DEA-efficient diet model has not been directly shown and the input data in this study had limited generalisability. Finally, using a country-specific database for GHGE was another strength. However, several limitations should be also mentioned. The results of this study including optimised diets should be interpreted with caution considering following limitations. First, the system boundary of the GHGE database was the production phase only. The diet-related GHGE could be underestimated due to without consideration of emission from post-production phases. Although GHGE in food system mainly comes from production phases, considering only production phase could affect in the calculation of optimised diet because some food item might have larger emission from post-production phase than other foods. In addition, diet-related GHGE was calculated based on the limited food information (i.e. GHGE values of 354 food items). Thus, the result for minimising diet-related GHGE should be interpret with caution. Second, the participants were not randomly sampled from the general Japanese population. This was a small convenient sample selected from the limited geographical area. In addition, participants were in relatively homogeneous social classes, were apparently healthy and were highly motivated to complete 4-d dietary records. These characteristics of the study population would be major drawbacks to investigate dietary patterns using DEA approaches and interpreting and extrapolating the results. Further studies are needed in a national representative sample, with a wider range of diets. Third, the dietary data were collected in winter (i.e. February to March) and relatively short-period (i.e. four-non-consecutive days). Previous studies have reported seasonal differences in intakes among Japanese adults^(51–53)^. Four-day records might not be enough for measuring long-term usual individual dietary intakes. Thus, this limited period for the survey might have produced some bias in assessing the usual intake and calculating optimised diets. Fourth, our model based on standardised EI may involve excess consumption of certain nutrients such as total fat. Further research is needed with considering excess EI. Fifth, the FBDG used in this study were not developed for Japan but for European countries. Similarly, NRF was also not developed for Japanese, although applicability of NRF was previously shown^(38)^ and used in a previous study with RDV based on Japanese Dietary Reference Intakes^(39)^. Applicability of the FBDG to Japan was unclear. For example, fish was included as dietary components to increase according to the FBDG used in the European context^(24)^, but fish intake in our Japanese population was already much higher than in these European populations^(24)^. Moreover, the EAT Lancer global reference diet also suggests differential FBDG for fish in east Asian/Pacific and European/Central Asian countries^(54)^. In contrast, the result that all modelled diets based on the FBDG had slightly improved nutritional quality suggests the alignment of favourable dietary patterns for Japanese and the European countries. In addition, the food group used in this study according to FBDG not much differ from those used in the previous study among Japanese^(12,29,45)^. Thus, benchmarking diets based on these FBDG could still be acceptable as alternative dietary patterns for Japanese. Sixth, prices for nearly half of the food items in food composition tables were substituted with the price of other food items, although the National Retail Price Survey covered prices of representative food items in Japan. Caution would be needed when interpreting the results of the optimised diets. However, the estimated dietary cost in the observed diet was not far from the mean national expenditure for foods per capita as calculated by the 2013 Family Income and Expenditure Survey (957 Japanese yen/person per d)^(55)^. Thus, the relatively large proportion of food items with substituted food prices for other food items might have not given a large effect on the result. Seventh, cultural acceptability and its indicator have not been formally defined. In this study, a higher similarity between modelled and observed diets was considered more culturally acceptable. Further discussion and studies needed to define it, because culturally acceptability is the major issue when optimising diets. Finally, the robustness of the DEA model might be impaired by the small sample size and a relatively large number of input and output variables (i.e. dietary components to increase and decrease). In addition, it was possible that the robustness was affected by not excluding participants with extraordinary intakes such as zero intakes or much larger intake than others, although non-parametric DEA model was used in this study. However, the focus of this study was not the calculated efficiency score, which would be more affected by the small sample size, but the calculation of the linear combination of DEA-efficient diets. In addition, excluding four nutrients from dietary components to increase and decrease provided similar results (data not shown), although some nutrients showed different results. Moreover, most of the participants who had extraordinary intake pattern would be excluded by the exclusion criterion based on EI. Furthermore, the optimised dietary intake pattern had some consistency in previous studies. Thus, it could not be concluded that the robustness of this study was low. To show a more robust result, further studies would be needed with a more representative and larger sample size and further selected or aggregated dietary components. In addition, more methodological research should be done on the application, robustness and sensitivity for DEA diet model.

In conclusion, this study suggests that alternative dietary intake patterns might be available for Japanese adults within the boundary of observed diets under the requirements to keep dietary similarity, improve nutritional quality, and reduce monetary cost and diet-related GHGE. The alternative diets proposed in this study could be the first step for future sustainable diets. However, based on the current analysis in healthy Japanese men and women, the relative level of improvement was rather small due to trade-offs between the different sustainable dimensions. These trade-offs underpin the need for modelling diets in representative groups for a wider range of East Asian/Pacific diets.

## Supplementary material

For supplementary material accompanying this paper visit https://doi.org/10.1017/S0007114522000095. click here to view supplementary material
