# Supplementary material for: Exploring culturally acceptable, nutritious, affordable and low climatic impact diet for Japanese diets: proof of concept of applying a new modelling approach using data envelopment analysis
Source: Br J Nutr. 2022 Jan 13;128(12):2438–52. doi: 10.1017/S0007114522000095 (PMC9723491; doi:10.1017/S0007114522000095)
Supplement: Supplementary file 1 [file S0007114522000095sup001.pdf]

Supplemental Table 1. Food group classification used in this study

Food group	Subgroup	Food items
Grains	Whole grains	Brown rice, whole grain flour, whole barley flour, whole grain noodle
	Refined grains	Well-milled rice, 70% milled rice, half milled rice, oats, bread, noodles, and flour
Potatoes		Potatoes, sweet potatoes, Japanese yam, French fries
Legumes, and nuts and seeds	Legumes	Adzuki beans, kidney beans, peas, soybeans and their products (tofu, natto)
	Nuts and seeds	Almond, chestnuts, walnuts, peanuts
Vegetables		Vegetables, mushrooms, and seaweeds
Fruit		Incl. 100% fruit juices
Fish/seafood		Horse mackerel, sardines, salmons, tunas, shrimps, crabs, squids, fish paste product
Meat		
		Incl. beef processed products such as corned beef
		Incl. pork processed products such as ham and bacon
		Horse, deer, rabbit, and other game meat
Eggs		Japanese quail's eggs, hen's eggs, chicken
Fish/seafood		Horse mackerel, sardines, salmons, tunas, shrimps, crabs, squids, fish paste product
Dairy products	Milk and dairy products	Ordinary liquid milk, whole milk powder, cream, yoghurt
	Cheese	Natural cheese, processed cheese
Alcoholic beverages		Beer, wine, sake, whiskey, mirin (a type of sake for cooking)
Sweetened beverages		50% fruit juice beverage (Valencia orange, pineapple), lactic acid bacteria beverages, fruit-flavoured and coloured drink, cola drink
Fats and oils	Solid fats	Lard, butters
	Oils	Olive oil, sesame oil, rapeseed oil, margarine, shortening, mayonnaise
Seasoning		Worcester sauces, soy sauces, common salt, vinegars, soup stocks, dressings, miso, roux
Sugar/confectionary		Brown sugar lump, sugar, honey, jams, manju (baked or steamed dough stuffed with filling), drops, rice crackers, bean jam bun, sponge cake, doughnuts, biscuits, milk chocolate, chewing gums, ice cream
Tea/coffee		Green teas, oolong tea, black tea, coffee, cocoa
Water		

Supplemental Table 2. Sex- and age-specific reference daily values used the calculation of the Nutrient-Rich Food (NRF) 15.3 score*

	Men			Women		
	18-29 y	30-49 y	50-69 y	18-29 y	30-49 y	50-69 y
Energy (kJ)	10460	10460	10460	8368	8368	8368
Qualifying nutrients						
Protein (g)†	60	60	60	50	50	50
Dietary fiber (g)‡	20	20	20	18	18	18
Vitamin A (μg RAE)†	850	900	850	650	700	700
Vitamin C (mg)†	100	100	100	100	100	100
Vitamin D (μg)§	5.5	5.5	5.5	5.5	5.5	5.5
Vitamin E (mg)§	6.5	6.5	6.5	6	6	6
Thiamine (mg)†	1.4	1.4	1.3	1.1	1.1	1
Riboflavin (mg)†	1.6	1.6	1.5	1.2	1.2	1.1
Vitamin B12 (μg)†	2.4	2.4	2.4	2.4	2.4	2.4
Mono-unsaturated fatty acids (g)||	27.8-55.6	27.8-55.6	27.8-55.6	22.2-44.4	22.2-44.4	22.2-44.4
Calcium (mg)†	800	650	700	650	650	650
Iron (mg)†	7	7.5	7.5	10.5	10.5	6.5
Zinc (mg)†	10	10	10	8	8	8
Folate (μg)†	240	240	240	240	240	240
Potassium (mg)‡	3000	3000	3000	2600	2600	2600
Disqualifying nutrients						
Saturated fat (g) ¶	19.4	19.4	19.4	15.6	15.6	15.6
Sodium (g NaCl equivalent/day)‡	<8	<8	<8	<7	<7	<7
Added sugar (g)**	31.25	31.25	31.25	25	25	25

*Values were derived from the Dietary Reference Intakes for Japanese, 2015 except for mono-unsaturated acid and added sugar (as shown below).

†Recommended Dietary Allowance, defined as "an estimate of the daily average dietary intake that satisfies the needs of most of the individuals belonging to a population (97–98%), on the basis of the distribution of the measured requirements of a study population[(1)]"

‡Tentative Dietary Goal for Preventing Lifestyle-related Diseases, defined as "the average daily-nutrient-intake level (or ranges) that Japanese should currently aim to consume primarily to prevent chronic diseases [(1)]"

§Adequate Intake, defined as "a recommended average daily-nutrient-intake level based on observed or experimentally determined approximations or estimates of nutrient intake by a group (or groups) of apparently healthy people that are assumed to be adequate[(1)]".

||Determined based on the recommendation in European countries (10-20% of energy)[(2)].

¶Determined based on the value of Tentative Dietary Goal for Preventing Lifestyle-related Diseases (7% of energy).

**Determined based on the World Health Organization's conditional recommendation (5% of energy) [(1)].

Supplemental Table 3. Comparison of food intake (g/10.460 MJ for men and g/8.386 MJ for women) in observed diet between participants with DEA-efficient diets and those with DEA-inefficient diets, 184 Japanese men and 185 women*

	Men					Women				
	With DEA-efficient diets * (n=74)		With DEA-inefficient diets (n=110)			With DEA-efficient diets * (n=71)		With DEA-inefficient diets (n=114)		
	Mean	SD	Mean	SD	P†	Mean	SD	Mean	SD	P†
Cereal	541	162	581	107	0.0001	395	105	410	77	0.003
Whole grain‡	10	57	2	11	<.0001	5	31	1	4	<.0001
Refined grain§	532	167	579	107	<.0001	390	112	409	76	0.0003
Potatoes	57	39	49	38	0.83	45	33	41	36	0.41
Legumes and nuts	79	71	53	42	<.0001	76	60	47	31	<.0001
Legumes‡	73	73	51	42	<.0001	72	59	44	30	<.0001
Nuts‡	6	14	3	5	<.0001	4	7	3	4	<.0001
Vegetables‡	309	146	274	92	<.0001	321	159	263	94	<.0001
Fruits‡	121	135	65	64	<.0001	118	113	86	77	0.0003
Meat, Fish, and Eggs	243	79	241	59	0.006	184	64	187	45	0.0007
Meat	114	64	116	51	0.03	67	50	89	36	0.003
Red and processed meat§	74	49	81	39	0.03	42	33	59	27	0.04
Beef	21	25	22	23	0.33	11	18	15	16	0.36
Pork	53	37	59	34	0.49	30	30	44	25	0.13
Other meat	0	0	0	1	<.0001	0	0	0	2	<.0001
Chicken	40	36	36	31	0.20	26	31	30	26	0.06
Fish‡	85	57	79	42	0.003	76	52	61	34	<.0001
Egg	43	24	46	22	0.43	40	24	37	22	0.40
Dairy products	118	123	78	73	<.0001	130	116	102	79	0.0003
Milk and other dairy products‡	114	123	74	74	<.0001	125	116	99	79	0.0002
Cheese	4	7	4	6	0.04	5	7	3	4	<.0001
Fat and oils	23	11	24	9	0.04	19	10	22	8	0.16
Fat	2	3	3	4	0.06	2	3	3	4	0.05
Oil	21	10	22	8	0.03	16	8	19	8	0.44
Sugar/confectionaries	54	46	53	33	0.002	61	41	65	32	0.02
Alcoholic beverages§	196	328	234	317	0.73	70	169	64	151	0.28
Tea/coffee	644	459	641	407	0.25	666	413	648	379	0.41
Sweetened beverages§	42	127	51	89	0.001	28	59	40	78	0.01
Seasonings	134	100	147	105	0.63	106	72	121	78	0.51
Water	607	486	509	302	<.0001	541	374	528	304	0.05

*'DEA-efficient diets' were identified as the diets having a higher multidimensional ratio of predefined 'dietary components to increase' per unit of 'dietary components to decrease' by using Data Envelopment Analysis. The rest of diets were defined as 'DEA-inefficient diets.'

†The t-test was performed. P < 0.05 was considered statistically significant.

‡Food group included as ' dietary components to increase' in Data Envelopment Analysis.

§Food group included as ' dietary components to decrease' in Data Envelopment Analysis.

Supplemental Table 4. Comparison of nutrition intake (per 10.460 MJ for men and per8.386 MJ for women) in observed diet between participants with DEA-efficient diets and those with DEA-inefficient diets, 184 Japanese men and 185 women*

	Men					Women				
	With DEA-efficient diets * (n=74)		With DEA-inefficient diets (n=110)			With DEA-efficient diets * (n=71)		With DEA-inefficient diets (n=114)		
	Mean	SD	Mean	SD	P	Mean	SD	Mean	SD	P
Protein (g)	88.4	14.4	86.0	10.6	0.004	73.8	12.8	70.2	7.5	<.0001
Total fat (g)	74.7	16.1	76.0	12.6	0.02	64.7	12.2	66.1	10.6	0.21
Saturated fat (g)	21.0	5.7	21.4	5.0	0.17	19.2	5.4	19.8	4.8	0.23
Unsaturated fatty acid (g)	43.0	10.4	43.6	8.3	0.03	35.9	7.0	37.6	6.6	0.53
Mono-unsaturated fatty acid (g)	27.2	7.3	28.0	6.1	0.12	22.6	5.0	24.4	4.6	0.45
Poly-unsaturated fatty acid (g)	15.8	4.1	15.5	2.8	0.0005	13.3	3.3	13.2	3.0	0.31
n-3 poly-unsaturated fatty acid (g)	13.1	3.6	12.8	2.5	0.0008	11.0	2.8	11.1	2.6	0.54
n-6 poly-unsaturated fatty acid (g)	2.7	0.9	2.7	1.0	0.50	2.3	1.0	2.1	0.7	0.003
Carbohydrate (g)	328	58	328	40	0.0002	268	30	268	24	0.04
Dietary fiber (g)	16.4	6.6	14.7	3.3	<.0001	16.0	4.8	13.7	3.0	<.0001
Sodium (mg)	4554	1128	4746	854	0.01	3848	1159	3876	737	<.0001
Potassium (mg)	3158	812	2770	545	0.0002	2996	721	2571	487	0.0002
Calcium (mg)	577	187	522	127	0.0002	615	219	495	112	<.0001
Magnesium (mg)	343	86	307	60	0.0005	319	85	274	51	<.0001
Phosphate (mg)	1274	208	1213	156	0.01	1136	209	1020	114	<.0001
Iron (mg)	10.0	2.4	8.9	1.7	0.002	9.1	2.4	7.9	1.5	<.0001
Zinc (mg)	10.5	2.7	10.0	1.6	<.0001	8.4	2.1	8.1	1.0	<.0001
Copper (mg)	1.5	0.3	1.3	0.2	0.01	1.3	0.3	1.1	0.2	<.0001
Vitamin A (μg RAE)	956	1370	498	192	<.0001	667	734	462	154	0.01
Vitamin D (μg)	9.0	5.6	8.5	5.6	0.96	8.5	6.0	6.7	4.4	<.0001
Vitamin E (mg)	8.9	2.5	8.0	1.5	<.0001	8.0	2.1	7.5	1.8	0.004
Vitamin K (μg)	270	112	245	104	0.45	292	144	241	104	0.19
Thiamin (mg)	1.2	0.3	1.2	0.3	0.28	1.0	0.2	1.0	0.2	0.002
Riboflavin (mg)	1.6	0.4	1.4	0.3	0.0006	1.4	0.4	1.3	0.2	0.22
Niacin (mg)	22.6	6.4	22.1	5.6	0.23	18.4	5.2	17.5	3.4	<.0001
Vitamin B6 (mg)	1.6	0.4	1.4	0.3	0.003	1.3	0.4	1.2	0.2	<.0001
Vitamin B12 (μg)	8.7	6.3	6.8	3.6	<.0001	7.2	5.4	5.5	3.3	<.0001
Folate (μg)	439	145	373	147	0.91	433	158	358	118	0.01
Pantothenic acid (mg)	7.3	1.4	6.6	1.1	0.04	6.4	1.6	5.8	0.8	<.0001
Vitamin C (mg)	132	58	107	42	0.003	136	59	113	42	0.001
Alcohol (g)	18.7	31.3	17.0	21.0	0.0002	5.2	10.2	4.0	7.6	0.005
Added sugar (g)	34.6	24.5	37.9	17.8	0.002	32.5	15.8	38.6	14.9	0.59

*‘DEA-efficient diets’ were identified as the diets having a higher multidimensional ratio of predefined ‘dietary components to increase’ per unit of ‘dietary components to decrease’ by using Data Envelopment Analysis. The rest of diets were defined as ‘DEA-inefficient diets.’

†The t-test was performed. P < 0.05 was considered statistically significant.

Supplemental Table 5. Nutrient intake (per 10.460 MJ for men and per 8.386 MJ for women) in observed diet and modelled diet among 184 Japanese men and 185 women*

	Men (n=184)												Women (n=185)											
	Observed		MaxA		MaxN		MinC		MinE		MIX		Observed		MaxA		MaxN		MinC		MinE		MIX	
	Mean	SD	Mean	SD	Mean	SD	Mean	SD	Mean	SD	Mean	SD	Mean	SD	Mean	SD	Mean	SD	Mean	SD	Mean	SD	Mean	SD
Protein (g)	86.9	12.3	88.0	10.6	94.0[a]	11.1	89.4[a]	10.8	88.7[a]	10.8	88.3[a]	10.2	86.9	12.3	72.5[a]	9.7	80.7[a]	10.3	71.8	10.1	72.7[a]	10.6	73.5[a]	9.8
Total fat (g)	75.5	14.1	75.1	11.6	76.4	10.7	74.6	12.0	73.2[a]	11.6	73.7[a]	11.4	75.5	14.1	66.4	10.1	63.6[a]	8.2	67.1[a]	9.9	65.6	9.8	66.2	9.9
Saturated fat (g)	21.2	5.3	21.2	4.2	21.2	3.8	21.3	4.4	20.3[a]	4.0	20.6[a]	4.0	21.2	5.3	19.5	4.2	18.2[a]	3.7	19.6	3.9	19.3	4.2	19.1	4.0
Unsaturated fatty acid (g)	43.3	9.2	42.8	7.2	43.5	6.8	42.1[a]	7.7	41.0[a]	7.5	41.8[a]	7.3	43.3	9.2	35.8[a]	5.2	36.7	4.9	37.0	5.1	36.7	5.3	36.7	5.0
Mono-unsaturated fatty acid (g)	27.7	6.6	27.1[a]	5.1	27.4	4.7	26.5[a]	5.4	25.4[a]	5.2	26.2[a]	5.1	27.7	6.6	22.5[a]	3.7	22.6[a]	3.3	22.8[a]	3.5	22.5[a]	3.7	22.7[a]	3.5
Poly-unsaturated fatty acid (g)	15.6	3.4	15.7	2.9	16.1	2.9	15.7	3.0	15.5	2.9	15.6	2.9	15.6	3.4	13.2	2.4	14.2[a]	2.5	14.1[a]	2.5	14.2[a]	2.6	13.9[a]	2.4
n-3 poly-unsaturated fatty acid (g)	2.7	0.9	2.7	0.7	3.0	0.7	2.8	0.7	2.9	0.7	2.8	0.7	2.7	0.9	2.2	0.7	2.2[a]	0.7	2.4[a]	0.7	2.3[a]	0.7	2.3[a]	0.7
n-6 poly-unsaturated fatty acid (g)	12.9	3.0	13.0	2.6	13.0[a]	2.5	12.8[a]	2.6	12.7[a]	2.6	12.8[a]	2.6	12.9	3.0	11.0	2.1	11.9	2.2	11.7[a]	2.1	11.9	2.3	11.6[a]	2.1
Carbohydrate (g)	328	48	331[a]	43.0	328	40.6	336[a]	43.3	343[a]	43.2	337[a]	42.4	328	48	267	23.5	271	20.4	269	23.2	272	24.3	269	23.2
Dietary fiber (g)	15.4	5.0	16.5[a]	5.3	19.8[a]	5.8	17.0[a]	5.3	18.9[a]	5.7	17.5[a]	5.2	15.4	5.0	15.2[a]	3.7	19.6[a]	4.6	15.9[a]	3.6	16.5[a]	3.5	16.1[a]	3.5
Sodium (mg)	4668	976	4435[a]	808	4194[a]	801	4368[a]	820	4417[a]	803	4379[a]	801	4668	976	3582[a]	841	3292[a]	868	3719[a]	857	3747[a]	847	3649[a]	851
Potassium (mg)	2926	690	3058[a]	615	3472[a]	613	3135[a]	587	3186[a]	588	3134[a]	580	2926	690	2929[a]	580	3687[a]	779	2904[a]	578	2997[a]	560	2944[a]	570
Calcium (mg)	544	156	566[a]	137	667[a]	154	593[a]	142	585[a]	143	569[a]	141	544	156	594[a]	173	709[a]	176	624[a]	160	641[a]	168	633[a]	159
Magnesium (mg)	321	74	332[a]	67	379[a]	69	350[a]	66	359[a]	67	348[a]	65	321	74	304[a]	64	371[a]	75	315[a]	63	325[a]	61	317[a]	61
Phosphate (mg)	1238	181	1262[a]	157	1386[a]	175	1299[a]	165	1321[a]	166	1291[a]	154	1238	181	1110[a]	166	1256[a]	171	1117[a]	159	1143[a]	169	1141[a]	157
Iron (mg)	9.3	2.1	9.8[a]	1.7	10.7[a]	1.8	9.8[a]	1.7	10.2[a]	1.8	9.9[a]	1.7	9.3	2.1	8.9[a]	1.7	10.6[a]	2.1	9.0[a]	1.7	9.1[a]	1.8	9.2[a]	1.7
Zinc (mg)	10.2	2.1	10.3	1.9	11.0[a]	2.0	10.3	1.9	10.2	1.8	10.3	1.8	10.2	2.1	8.1	1.5	9.1[a]	1.5	8.1	1.4	8.2	1.5	8.2	1.4
Copper (mg)	1.4	0.3	1.5[a]	0.2	1.5[a]	0.2	1.5[a]	0.2	1.5[a]	0.2	1.5[a]	0.2	1.4	0.3	1.2[a]	0.2	1.5[a]	0.3	1.3[a]	0.2	1.3[a]	0.2	1.3[a]	0.2
Vitamin A (µg RAE)	683	906	840[a]	933	902[a]	870	775[a]	885	760[a]	886	846[a]	879	683	906	633[a]	476	713[a]	461	608[a]	465	623[a]	463	689[a]	464
Vitamin D (µg)	8.7	5.6	8.5	4.0	9.3	3.9	9.8[a]	3.9	10.2[a]	3.9	9.7[a]	3.9	8.7	5.6	8.1[a]	4.2	8.6[a]	4.0	7.6	4.4	8.9[a]	5.2	8.8[a]	4.7
Vitamin E (mg)	8.4	2.0	8.9[a]	1.9	9.7[a]	1.9	9.1[a]	2.0	9.4[a]	1.9	9.4[a]	2.0	8.4	2.0	8.0[a]	1.5	8.9[a]	1.7	8.4[a]	1.7	8.5[a]	1.7	8.3[a]	1.6
Vitamin K (µg)	255	107	271[a]	90.2	348[a]	117.9	291[a]	96.6	330[a]	111.7	295[a]	94.7	255	107	288[a]	110.2	384[a]	126.3	302[a]	107.6	292[a]	107.4	303[a]	104.4
Thiamine (mg)	1.2	0.3	1.1	0.2	1.3[a]	0.2	1.1	0.2	1.2	0.2	1.2	0.2	1.2	0.3	0.9[a]	0.2	1.0[a]	0.2	0.9[a]	0.2	1.0	0.2	0.9	0.2
Riboflavin (mg)	1.5	0.4	1.5[a]	0.3	1.7[a]	0.3	1.6[a]	0.3	1.6[a]	0.3	1.6[a]	0.3	1.5	0.4	1.4[a]	0.3	1.7[a]	0.4	1.4[a]	0.3	1.4[a]	0.3	1.4[a]	0.3
Niacin (mg)	22.3	5.9	21.9	4.7	23.9[a]	4.5	21.9	4.6	22.6	4.5	22.4	4.5	22.3	5.9	18.2	3.9	22.0[a]	4.7	17.0[a]	4.1	18.0	3.7	17.6	3.9
Vitamin B6 (mg)	1.5	0.4	1.6[a]	0.3	1.8[a]	0.3	1.6[a]	0.3	1.7[a]	0.3	1.7[a]	0.3	1.5	0.4	1.3[a]	0.3	1.7[a]	0.4	1.3[a]	0.3	1.3[a]	0.3	1.3[a]	0.3
Vitamin B12 (µg)	7.6	4.9	8.2[a]	4.5	9.6[a]	4.6	8.6[a]	4.4	8.9[a]	4.5	8.9[a]	4.5	7.6	4.9	6.7[a]	3.9	6.4	3.9	6.6[a]	3.9	6.9[a]	4.0	6.8[a]	3.8
Folate (µg)	399	150	428[a]	110	502[a]	118	421[a]	117	451[a]	117	431[a]	111	399	150	411[a]	112	522[a]	132	412[a]	112	417[a]	116	430[a]	107
Pantothenic acid (mg)	6.9	1.3	7.1[a]	1.1	8.1[a]	1.2	7.4[a]	1.0	7.8[a]	1.2	7.5[a]	1.0	6.9	1.3	6.3[a]	1.2	7.8[a]	1.6	6.3[a]	1.1	6.3[a]	1.2	6.4[a]	1.1
Vitamin C (mg)	117	50	129[a]	43	155[a]	46	129[a]	45	139[a]	46	131[a]	43	117	50	126	44	173[a]	53	129[a]	45	140[a]	46	134[a]	42
Alcohol (g)	17.7	25.6	16.1[a]	24.0	14.1[a]	22.7	13.3[a]	22.4	12.1[a]	22.7	14.5[a]	22.9	17.7	25.6	3.8[a]	7.6	2.7[a]	6.7	2.6[a]	6.7	2.6[a]	6.7	2.9[a]	6.8
Added sugar (g)	36.6	20.7	33.5[a]	18.1	30.8[a]	16.3	33.7[a]	18.6	30.2[a]	18.3	31.1[a]	16.9	36.6	20.7	30.8[a]	11.4	26.6[a]	11.1	30.0[a]	11.0	28.7[a]	11.2	28.7[a]	10.8

MaxA, the modelled diet with the most culturally acceptable (i.e. smallest change in consumption of 21 food groups from observed diet); MaxN, the modelled diet with highest nutritional quality assessed by Nutrient-Rich Food Index 15.3 score; MinC, the modelled diet with least monetary cost of diet; MinE, the modelled diet with the least diet-related greenhouse gas emissions; MIX, the modelled diet that all selected indicators (maximize cultural acceptability and nutritional quality and minimize diet-related GHGE and monetary cost) were equally considered.

*Values are mean and SD of nutrient intake in the observed diet and modelled diet.

Appendix A, Description of basic Data Envelopment Analysis models

Input-oriented and output-oriented Banker, Charnes and Cooper (BCC) models[3] for Data Envelopment Analysis (DEA) was used. Following the description of the DEA model was according to the previous study by Kanellopoulos, et al [4].

Input-oriented DEA:

$$min\left\{\theta - \varepsilon\left(\sum_{\iota} s_i^{in} + \sum_{j} s_j^{ot}\right)\right\} \qquad \theta \in \{0 \ldots 1\}$$

s.t.:

$$\sum_{k} y_{jk}\lambda_k - y_j^0 - s_j^{ot} = 0 \qquad \forall\, j$$

$$\sum_{k} x_{ik}\lambda_k - \theta x_i^0 + s_i^{in} = 0 \qquad \forall\, i$$

$$\sum_{k} \lambda_k = 1$$

$$\lambda_k, s_j^{ot}, s_i^{in} \geq 0 \qquad \forall\, k, j, i$$

Output-oriented DEA:

$$max\left\{\varphi + \varepsilon\left(\sum_{\iota} s_i^{in} + \sum_{j} s_j^{ot}\right)\right\}$$

s.t.:

$$\sum_{k} y_{jk}\lambda_k - \varphi y_j^0 - s_j^{ot} = 0 \qquad \forall\, j$$

$$\sum_{k} x_{ik}\lambda_k - x_i^0 + s_i^{in} = 0 \qquad \forall\, i$$

$$\sum_{k} \lambda_k = 1$$

$$\lambda_k, s_j^{ot}, s_i^{in} \geq 0 \qquad \forall\, k, j, i$$

Where θ is the efficiency score of the evaluated diet calculated with the input-oriented DEA model (DEA-efficient diets get the value of 1), λ_k is a decision variable and the weight of diet k in the alternative of the evaluated diet, s_j^{ot} is the slack decision variable capturing the

deviation between the intake of 'dietary components to increase' food or nutrient j of the alternative diet and that of the current diet, s_i^{in} is the slack decision variable that captures the deviation between the intake of 'dietary components to decrease' nutrient i of the current diet and that of the alternative diet, ε is a marginal (i.e. very small) positive number, x_{ik} is intake of 'dietary components to decrease' food or nutrient i in diet k, y_{jk} is intake of 'dietary components to increase' food or nutrient j in diet k, x_i^0 is intake of 'dietary components to decrease' nutrient i in the evaluated diet, and y_j^0 is intake of 'dietary components to increase' for or nutrient j in the evaluated diet.

φ is the efficiency score of the evaluated diet calculated with the output-oriented DEA model. The higher the value of φ the higher the efficiency of the evaluated diet. To normalize the efficiency scores of the output-oriented DEA to values from 0 (i.e. lowest efficiency) to 1 (i.e. highest efficiency), efficiency scores were reported as φ^{-1}. Both the input-oriented DEA and the output-oriented DEA models were solved in two stages following Cooper et al. [3]

In the benchmarking process of diets, the efficiency scores θ $(0 \leq \theta \leq 1)$ and φ^{-1} $(0 \leq \varphi^{-1} \leq 1)$ were calculated[4], respectively, for each individual following the method by Cooper, et al [3]. As a result of the analysis, the diet with $\theta = 1$ or $\varphi^{-1} = 1$ was identified as 'DEA-efficient diets.' It was confirmed that the same participants were identified as with 'DEA-efficient diets' in both input- and output-oriented DEA models.

Appendix B, Sets, indices, model parameters, and decision variables for Appendix C

Table B1. Sets and indices

Index	Description
l	Index for different objectives
k	Index of modelled diets of individuals
m	Nutrients used to calculate the Nutrient Rich Food index score
j	Food groups and nutrients included in the benchmarking with DEA as dietary components to increase
i	Food groups and nutrients included in the benchmarking with DEA as dietary components to decrease
f	Food groups for calculating deviation between food consumption in modelled diets and the observed diets

Table B2: Model parameters

Parameter	Description	Units
$cost_k$	The monetary cost of diet *k*	Japanese yen
$GHGE_k$	Diet-related greenhouse gas emissions of diet *k*	gram CO_2-eq
$RDVL_m$	The lower limit of the reference value. For the qualifying nutrients in Nutrient Rich Food (NRF) 15.3 including protein, dietary fibre, vitamin A, B12, C, D, E, thiamine, riboflavin,calcium, iron, zinc, folate, potassium, $RDVL_m$ was the reference value of each nutrient. For monosaturated fatty acid, $RDVL_m$ was its lower limit (i.e. 10% energy). For the disqualifying nutrients in NRF 15.3, $RDVL_m$=0 was assigned.	gram, mg or µg
$RDVU_m$	The upper limit of the reference value. For the disqualifying nutrients in NRF 15.3 including saturated fatty acid, sodium, and added sugar, $RDVU_m$ was reference value of each nutrient. For mono-saturated fatty acid, $RDVU_m$ was its upper limit (i.e. 20% energy). For the other qualifying nutrients in NRF 15.3, $RDVU_m$=0 was assigned.	gram, mg or µg
V_l^{max}	The maximum observed value for objective *l*	gram for V_A^{max}, no unit for V_N^{max}, Japanese yen for V_C^{max}, g CO_2-eq for V_E^{max}
w_l	The importance weight of objective function *l*	-
d_f^+	The positive deviations of the intakes of food group f between the calculated and the current diets	gram
d_f^-	The negative deviations of the intakes of food group f between the calculated and the current diets	gram
$Nutr_{m,k}$	The observed intakes for nutrient *m* included in NRF 15.3 score in diet *k*	gram, mg or µg
du_m^+	The positive deviation of the intake of nutrient *m* in the modelled diet from the reference value	gram, mg or µg
du_m^-	The negative deviation of the intake of nutrient *m* in the modelled diet from the reference value	gram, mg or µg
dl_m^+	The positive deviation of the intake of nutrient *m* in the modelled diet from the reference value	gram, mg or µg
dl_m^-	The negative deviation of the intake of nutrient *m* in the modelled diet from the reference value	gram, mg or µg
FG_{fk}	The intake of food group *f* in diet *k*	gram
FG_f^0	The consumption of food group *f* in the evaluated diet	gram
y_{jk}	The amount of intake of dietary components to increase *j* in the diet *k*	gram
y_j^0	The amount of intake of dietary components to increase *j* in the diet of the evaluated diet	gram
x_{ik}	The amount of intake of dietary components to decrease *i* in the diet *k*	gram
x_i^0	The amount of intake of dietary components to decrease *i* in the evaluated diet	gram

Table B3: Decision variables

Variable	Description	Units
λ_k	The proportion of diet k in the modelled diet	-
V_l	Value of objective l, namely, $V_{acceptability}$ the total deviation of food item intakes between modelled and observed diet, V_{NRF} the NRF score of the modelled diet, V_{cost} the total cost of the modelled diet and V_{GHGE} the total greenhouse gas emissions of the modelled diet	gram for $V_{accecibility}$, no unit for V_{NRF}, Japanese yen for V_{cost}, g CO_2-eq for V_{GHGE}

Table B2. Model parameters

Parameter	Description	Units
cst_k	Monetary cost of diet *k*	Japanese yen
ems_k	Diet-related greenhouse gas emissions of diet *k*	gram CO_2-eq
$RDVL_m$	Lower limit of the reference value. For the qualifying nutrients in Nutrient Rich Food (NRF) 15.3 including protein, dietary fibre, vitamin A, B1, B2, B12, C, D, E, calcium, iron, zinc, folate, potassium, $RDVL_m$ was the reference value of each nutrient. For monosaturated fatty acid, $RDVL_m$ was its lower limit (i.e. 10% energy). For the disqualifying nutrients in NRF 15.3, $RDVL_m$=0 was assigned.	gram, mg or µg
$RDVU_m$	Upper limit of the reference value. For the disqualifying nutrients in NRF 15.3 including saturated fatty acid, sodium, and added sugar, $RDVU_m$ was reference value of each nutrient. For mono-saturated fatty acid, $RDVU_m$ was its upper limit (i.e. 20% energy). For the other qualifying nutrients in NRF 15.3, $RDVU_m$=0 was assigned.	gram, mg or µg
F_g^{min}	The minimum observed value for objective *g*	gram for V_A^{min} and V_A^{max}, no unit for V_N^{min} and V_N^{max},
F_g^{max}	The maximum observed value for objective *g*	Japanese yen for V_C^{min} and V_C^{max}, g CO_2-eq for V_E^{min} and V_E^{max}
w_g	The importance weight of objective function *g*	-
d_f^+	The positive deviations of the intakes of food group f between the calculated and the current diets	gram
d_f^-	The negative deviations of the intakes of food group f between the calculated and the current diets	gram
$NI_{m,k}$	The observed intakes for nutrient *m* included in NRF 15.3 score in diet *k*	gram, mg or µg
du_m^+	The positive deviation of the intake of nutrient *m* in the modelled diet from the reference value	gram, mg or µg
du_m^-	The negative deviation of the intake of nutrient *m* in the modelled diet from the reference value	gram, mg or µg
dl_m^+	The positive deviation of the intake of nutrient *m* in the modelled diet from the reference value	gram, mg or µg
dl_m^-	The negative deviation of the intake of nutrient *m* in the modelled diet from the reference value	gram, mg or µg
FG_{fk}	The intake of food group *f* in diet *k*	gram
FG_f^0	The consumption of food group *f* in the evaluated diet	gram
y_{jk}	The amount of intake of "dietary components to increase" food or nutrient *j* in the diet *k*	gram
y_j^0	The amount of intake of "dietary components to increase" food or nutrient *j* in the diet of the evaluated diet	gram
x_{ik}	The amount of intake of "dietary components to decrease" food or nutrient *i* in the diet *k*	gram
x_i^0	The amount of intake of "dietary components to decrease" food or nutrient *i* in the evaluated diet	gram

Table B3. Decision variables

Variable	Description	Units
λ_k	Share of diet k in the modelled diet	-
V_g	Value of objective *g*, i.e. F_P the total deviation of food item intakes between modelled and observed diet, F_N the NRF score of the modelled diet, F_C the total cost of the modelled diet and F_G the total greenhouse gas emissions of the modelled diet	gram for V_A, no unit for V_N, Japanese yen for V_C, g CO_2-eq for V_E
D	The aggregated objective function	-

Appendix C: Formulas

$$minimise \left\{ w_{acceptability} \times \left(\frac{V_{acceptability}}{V_{acceptability}^{max}}\right) - w_{NRF} \times \left(\frac{V_{NRF}}{V_{NRF}^{max}}\right) + w_{cost} \times \left(\frac{V_{cost}}{V_{cost}^{max}}\right) + w_{GHGE} \times \left(\frac{V_{GHGE}}{V_{GHGE}^{max}}\right) \right\} \quad (1)$$

Subject to:

$$V_A = \sum_f d_f^+ + d_f^- \quad (2)$$

$$V_N = 1500 - \sum_m \left(\frac{du_q^-}{RDV_{L_m}} \times 100 - \frac{dl_m^+}{RDV_{Um}} \times 100\right) \quad (3)$$

$$V_C = \sum_k cost_k \lambda_k \quad (4)$$

$$V_E = \sum_k GHGE_k \lambda_k \quad (5)$$

$$\sum_k \lambda_k FG_{f,k} + df_f^- - df_f^+ = FG_f^0 \qquad \forall f \quad (6)$$

$$\sum_k Nutr_{m,k} \lambda_k + du_m^- - du_m^+ = RDVL_m \qquad \forall m \;\; | \, RDVL_m > 0 \quad (7)$$

$$\sum_k Nutr_{m,k} \lambda_k + dl_m^- - dl_m^+ = RDVU_m \qquad \forall m \;\; | \, RDVU_m > 0 \quad (8)$$

$$\sum_k \lambda_k y_{jk} - y_j^0 \geq 0 \qquad \forall j \quad (9)$$

$$\sum_k \lambda_k x_{ik} - x_i^0 \leq 0 \qquad \forall i \quad (10)$$

$$\sum_k \lambda_k = 1 \quad (11)$$

$$\lambda_k \geq 0 \qquad \forall k \quad (12)$$

Appendix D: Detailed method for calculating optimized diets using a DEA diet model

First, observed diets were benchmarked to identify so-called 'DEA-efficient diets' defined as diets having a higher multidimensional ratio of intakes of 'dietary components (i.e., foods and nutrients) to increase' per unit of intakes of 'dietary components to decrease'(5). Intakes of dietary components to increase and those to decrease are interpreted as the inputs and outputs of a conventional DEA model(4), respectively. The rest of the diets not identified as so-called 'DEA-inefficient diets' (Step1 in Fig. 1). In the benchmarking process, input- and output-oriented DEA model (Appendix A in the Supplemental Material) was used to calculate multidimensional ratio according to Banker, Charnes and Cooper models (3). In this process, the efficiency score θ was calculated for each diet. As a result of the analysis, the diet with $\theta = 1$ was identified as DEA-efficient diets. The dietary components to increase and those to the decrease were selected from the previously defined food-based dietary guidelines(5,6). The dietary components to increase included fruits, vegetables, legumes, nuts/seeds, milk/cream/yoghurt, fish/seafood, and whole grains. The dietary components to decrease included red and processed meat, refined grains, sweetened beverages, and ethanol (as a proxy of alcoholic beverage). In addition, vitamin A (as a nutrient to increase), sodium, and added sugar (as nutrients to decrease) were included to be safeguarded, i.e., to avoid unwanted decrease or increase intakes of these nutrients in modelled diets. Zero intakes for dietary components to increase and those to the decrease were replaced by non-zero values, i.e., the lowest non-zero intake among the participants divided by two. This replacement was applied only in the benchmarking analysis to avoid zero values in denominators of the multidimensional ratio of intake.

Next, for the participants with DEA-inefficient diets, the alternative diets were calculated as linear combinations of observed DEA-efficient diets (Step 2 in Fig. 1). For example, an alternative diet for DEA-inefficient diets was calculated as follows:

$$\mathrm{Id}' = \sum \mathrm{Ed}_k \cdot \lambda_k \qquad \textit{s.t.}\ \ 0 \le \lambda_k,\ \ \sum \lambda_k = 1$$

where Id' is an alternative diet for DEA-inefficient diet Id, Ed_k is the DEA-efficient diet by a participant k, λ_k is the proportion of Ed_k in the alternative diet. Thus, combination of food intakes in the diet Ed_k is reflected in the alternative diet in the proportion λ_k. For a simple example, a combination of food intakes in an alternative diet for Id_1 is calculated as a sum of the combination

of food intakes in $Ed_1 \times 0.3$ and that in $Ed_2 \times 0.7$, when $\lambda_1 = 0.3$ and $\lambda_2 = 0.7$ was obtained as a solution for λ_k resulted from the calculation. In this step, the alternative diets were set to comprise of larger or equal intakes of dietary components to increase and smaller intakes of dietary components to decrease compared to each observed DEA-ineffieicnt diet. This constraint was imposed to make the modelled diets arrive at a better diet concerning the intakes of dietary components to increase and those to decrease.

By changing the proportion of each DEA-efficient diet (λ_k) in the combinations, modelled diets that optimized a certain indicator were obtained within the boundary of observed DEA-efficient diets under the requirement to improve intakes of dietary components to increase and those to decrease (Step 2 and 3 in Fig. 1). The proportion of each DEA-efficient diet in the combinations was obtained by solving the linear programming (Appendix B and C) for three types of models: maximum/minimum models (Step 4 in Fig. 1), optimal model (Step 5 in Fig. 1), and trade-off models (Step 6 in Fig. 5). Minimum/maximum models aimed to obtain diets achieving one of four goals, separately: (1) maximum cultural acceptability ($MAX_{acceptability}$), (2) maximum NRF 15.3 score (MAX_{NRF}), (3) minimum monetary cost (MIN_{cost}), (4) minimum diet-related GHGE (MIN_{GHGE}). There was no constraint introduced in each minimum/maximum model to the variables other than target variables. The optimal model considered all four goals in one model simultaneously (OPT_{all}). Trade-off models were aimed to examine the trade-offs between the goals, especially MAX_{NRF}, MIN_{cost} and MIN_{GHGE}.

To compose the linear programming model, firstly, four decision variables were formulated: $V_{acceptability}$ for cultural acceptability expressed as the total deviation of food intakes between modelled diets and observed diets (formulas (2) and (6) in Appendix C); V_{NRF} for NRF 15.3 score in the modelled diets (formulas (3), (7) and (8) in Appendix C); V_{cost} for the monetary cost of modelled diets (formula (4) in Appendix C); V_{GHGE} for diet-related GHGE in modelled diets (formula (5) in Appendix C). Nutrient intakes, monetary cost, diet-related GHGE in modelled diets were calculated as linear combinations of DEA-efficient diets using the proportion of each DEA-efficient diet (λ_k). NRF 15.3 scores in modelled diets were then obtained based on the calculated nutrient intakes in the modelled diet. To standardized the unit of the objectives, each decision variable was rescaled[5] by dividing with maximum observed value ($V^{max}_{acceptability}$, V^{max}_{NRF}, V^{max}_{cost},

and V_{GHGE}^{max}, respectively). The aggregated objective function was then formulated as follows.

Objective function:

Minimise $\{w_{acceptability} \times V_{acceptability}/V_{acceptability}^{max} - w_{NRF} \times V_{NRF}/V_{NRF}^{max} + w_{cost} \times V_{cost}/V_{cost}^{max} + w_{GHGE} \times V_{GHGE}/V_{GHGE}^{max}\}$………………..(formula (1) in Appendix C)

In maximum/minimum models, full weight was assigned for the targeted goal and zero weight was assigned for the rest. For example, weights in the MAX$_{acceptability}$ model were $w_{acceptability}$ = 1 and w_{NRF} = w_{cost} = w_{GHGE} = 0. In the OPT$_{all}$ model, same weights (i.e., 0.25) were assigned for all goals. In trade-off models, nine intermediate modelled diets between MAX$_{NRF}$ and MIN$_{cost}$ were calculated by applying the stepwise change of the weights by 10% (w_{NRF}/w_{cost} = 0.1/0.9, 0.2/0.8, 0.3/0.7, ..., 0.9/0.1). The intermediate modelled diets between MAX$_{NRF}$ *vs.* MIN$_{GHGE}$ and MIN$_{cost}$ *vs.* MIN$_{GHGE}$ were calculated in the same way.

Reference

1. Ministry of Health Labour and Welfare (2015) *Dietary Reference Intakes for Japanese, 2015.* .
2. Food and Agriculture Organization (2008) *Fats and fatty acids in human nutrition. Proceedings of the Joint FAO/WHO Expert Consultation. Ann. Nutr. Metab.*, vol. 55. Geneva, Switzerland: .
3. Cooper WW, Seiford LM & Tone K (2007) *Data Envelopment Analysis*. New York: Springer.
4. Kanellopoulos A, Gerdessen JC, Ivancic A, et al. (2020) Designing healthier and acceptable diets using data envelopment analysis. *Public Health Nutr.* **23**, 2290–2302.
5. Mertens E, Kuijsten A, Kanellopoulos A, et al. (2020) Improving health and environmental sustainability of European diets using a benchmarking approach. *Public Health Nutr.*, 1-11. Online ahead of print.
6. Mertens E, Kuijsten A, Dofková M, et al. (2019) Geographic and socioeconomic diversity of food and nutrient intakes: a comparison of four European countries. *Eur. J. Nutr.* **58**, 1475–1493.
